# Quantifying arsenic-binding affinities of ArsR proteins via biomimetic self-assembly

**DOI:** 10.3389/fmicb.2026.1846435

**Published:** 2026-05-22

**Authors:** Liang Cui, Xiaobo Zhang, Xiaohui Sun, Yi Yang, Bitong Zhu, Shasha Wang, RuoXin Lin, Chungui Zhao, Guangya Zhang, Jian Chen, Suping Yang

**Affiliations:** 1 Department of Bioengineering and Biotechnology, College of Chemical Engineering, Huaqiao University, Xiamen, Fujian, China; 2 Key Laboratory of Marine Genetic Resources, Third Institute of Oceanography, Ministry of Natural Resources, Xiamen, Fujian, China; 3 Fujian Ocean Innovation Center, Xiamen, Fujian, China; 4 The Second Hospital and Clinical Medicine School, Lanzhou University, Lanzhou, China; 5 Department of Cellular Biology and Pharmacology, Herbert Wertheim College of Medicine, Florida International University, Miami, FL, United States

**Keywords:** arsenic, ArsR (As(III)-binding transcriptional repressor protein), *Rhodopseudomonas palustris* CGA009, solid-phase arsenic adsorbent, SpyCatcher/SpyTag

## Abstract

ArsR, an As(III)-binding transcriptional repressor protein, plays a critical role in arsenic (As) detoxification by selectively binding As(III) and regulating cellular responses. Recent studies have revealed that ArsRs exhibit broad diversity in their arsenic recognition and binding sites. Multiple ArsRs are often found in highly As-resistant microbes, allowing for coping with complicated arsenical stresses. However, quantitative assessments of ArsR binding affinities and their contributions to arsenic resistance remain limited due to methodological challenges. In this study, we developed a novel biomimetic self-assembly approach, applied in a protein purification-free manner after initial validation, to quantify the arsenic binding affinity of ArsR-As interaction. ArsR was immobilized on the surface of biosilica spheres (S) via the covalently cross-link self-assembly of ArsR-SpyTag and ELP-SpyCatcher@SiO_2_, created a novel solid-phase arsenic adsorbent (S-ArsR) for precise binding affinity measurements. Using this technological platform, we characterized nine diverse ArsR homologs (RpArsR) from the highly arsenic-resistant bacterium *Rhodopseudomonas palustris* CGA009. RpArsR1 and RpArsR2 exhibited the highest affinity constants (*K*_*A*_ > 10^7^ M) for As(III), with binding affinity influenced by both cysteine content and structural context. Phylogenetic analysis clustered nine RpArsRs into three distinct subgroups (I, III, and IV), with binding affinities ranked as III > I > IV. These results reveal functional diversity in As(III)-binding behavior among ArsR homologs and provide a quantitative framework for comparing their binding properties. Notably, RpArsR2 significantly lowered As(III) accumulation in plants, highlighting its bioremediation potential. Our work enables a purification-free application strategy after initial validation and offers broad applicability for analyzing various protein-ligand interactions. It also provides a new strategy for developing highly selective arsenic adsorbents for environmental bioremediation.

## Introduction

Arsenic (As), a naturally occurring element prevalent in soil and groundwater, poses a significant threat to human health and ecosystems due to its bioaccumulation effect and toxicity (Zhu et al., 2014; Podgorski and Berg, 2020). In response to arsenic exposure, many microorganisms have evolved sophisticated detoxification systems, with some species even capable of utilizing arsenic compounds as energy sources. This microbial adaptability has led to the development of bioremediation strategies that offer efficient, cost-effective, and environmentally sustainable solutions for arsenic pollution mitigation. The arsenic detoxification system in microbes is primarily governed by arsenic resistance (*ars*) operons, which typically consist of arsenic related functional genes, such as *arsRBC*, *arsRDABC*, or *arsRM* (Andres and Bertin, 2016). The key component of this operon is ArsR protein, an As(III)-specific transcriptional repressor encoded by the *arsR* gene (Dunivin et al., 2019). Upon As(III) exposure, ArsR binds As(III) and undergoes conformational changes that lead to the derepression of *ars* genes, facilitating arsenic efflux, reduction, or methylation.

ArsR functions as a regulator that not only controls the transcriptional expression of *ars* genes, but also exhibits autoregulation (Chen et al., 2014), and can modulate the expression of non-arsenic-related genes (Kang et al., 2016). In addition, new types of ArsR repressors continue to be identified, governing distinct *ars* operons (Zhang et al., 2025). ArsR is one of the most extensively studied members of the ArsR/SmtB family, a group of transcriptional regulators widely distributed among prokaryotes. This family plays an important role in metal ion homeostasis and environmental adaptation. Structurally, ArsR typically consists of two core functional domains. The N-terminal DNA-binding domain contains a conserved helix-turn-helix (HTH) motif that binds specifically to operator sequences upstream of the *ars* operon, repressing transcription under arsenic-free conditions. The C-terminal metal-binding domain coordinates metal or metalloid ions, particularly As(III), via thiol groups of conserved cysteine residues, usually arranged in pairs or clusters.

While ArsR proteins share a common regulatory function, they exhibit substantial diversity in their amino acid sequences, metal-binding sites, and regulatory behaviors, reflecting their adaptation to a wide range of environmental conditions. Evolutionary analyses of the ArsR/SmtB family have revealed that different ArsR homologs have evolved distinct metal-binding motifs with variable numbers and arrangements of cysteine residues, allowing them to selectively sense specific metal or metalloid ions, such as As(III), antimonite [Sb(III)] and methylarsenite [MAs(III)].

To date, numerous distinct ArsR homologs have been characterized, reflecting the evolutionary diversity of arsenic-binding sites within this family of metalloregulators. For example, EcArsR, AfArsR, and CgArsR have been identified in *Escherichia coli* plasmid R773, *Acidithiobacillus ferrooxidans*, and *Corynebacterium glutamicum* ATCC13032, respectively. These proteins all respond to As(III), but exhibit distinct three-coordinate As(III)-binding motifs formed by cysteine (Cys) residues. These motifs are structurally conserved but vary in position: Cys32, Cys34, and Cys37 in EcArsR; Cys95, Cys96, and Cys102 in AfArsR; and Cys15, Cys16, and Cys55 in CgArsR. Another unique example is SpArsR, identified in *Shewanella putrefaciens* 200, which functions as a MAs(III)-specific repressor. SpArsR features a two-coordinate MAs(III) binding motif, composed of Cys101 and Cys102 (analogous to Cys95 and Cys96 in AfArsR). The absence of a third cysteine renders SpArsR unresponsive to inorganic As(III), highlighting its evolved selectivity for organoarsenical. The ability of ArsR to specifically recognize and bind arsenic has made it a widely utilized component in the development of arsenic biosensors and arsenic-accumulating systems for bioremediation.

In addition, many microorganisms harbor multiple *arsR* genes and diverse arsenic resistance determinants, enabling them to thrive in complex, arsenic-contaminated environments (Yang et al., 2010; Rawle et al., 2019; Rawle et al., 2021). This genomic arrangement provides regulatory flexibility and enables microbes to fine-tune their responses to fluctuating arsenic concentrations and mixed metal stresses in the environment. For example, in *Pseudomonas putida* KT2440, the genome contains two paralogous *ars* operons (*ars*1 and *ars*2), each regulated by its respective ArsR protein., and their expression regulated by temperature (Paez-Espino et al., 2015). *Agrobacterium tumefaciens* 5A has four ArsRs, which not only mediate arsenic resistance but also regulate non-arsenic metabolic genes (such as *pstS1* and *phoB1*), but their cross-regulatory mechanisms remain unclear (Rawle et al., 2019).

Our previous study showed that *Rhodopseudomonas palustris* CGA009, a highly arsenic-resistance bacterium, has at least three *ars* operons (*arsR_1_C_1_BH*, *arsR_2_C_3_C_2_ acr3*, *arsR_4_M*) and four ArsRs on genome, enabling As(V) reduction, As(III) efflux and As(III) methylation, respectively (Zhao et al., 2015), and ArsR2 displayed robust As (III) resistance (Ke et al., 2018). However, despite its widespread distribution, the binding affinities between different ArsR homologs and arsenic species have not been systematically compared or quantitatively analyzed. The individual contributions of these ArsRs to cellular As resistance and their potential cross-interaction mechanism remain poorly understood.

There is a clear need to establish precise and standardized methods for evaluating the As–ArsR interaction. Such quantitative insight is essential for understanding the molecular mechanisms underlying differential arsenic resistance and for optimizing ArsR-based applications. Spectrofluorometry (a homogeneous system) has been applied to examine the binding affinity between As and As-related proteins (Chen and Rosen, 1997; Chen et al., 2014), but the fluorescent quench signal of RpArsRs of *R. palustris* CGA009 had little changes (< 8%) even at the molar ratio of ArsR to As (III) reached 1:20, precluding reliable affinity measurements. HPLC-ICP-MS can measure As(III)-metallothionein coordination numbers but cannot determine binding constants and requires substrate-product separation (Jiang et al., 2003), and both methods required protein purification.

In this study, we developed a quantitative approach, applied in a protein purification-free manner after initial validation, to assess the arsenic-binding affinities of diverse RpArsRs and to explore their functional diversity and potential in biotechnological applications. Biosilica spheres (ELP-SpyCatcher@SiO_2_) were first synthesized via biomimetic silicification, followed by self-assembly of RpArsR proteins onto their surfaces using SpyTag/SpyCatcher site-specific recognition, creating a solid-phase arsenic adsorbent (S-RpArsR). It eliminates the need for purified RpArsR proteins, instead utilizing crude protein extracts to synthesize S-RpArsR. This technology enabled direct quantification of protein-As(III) binding affinities on the S-RpArsR surface. Using this technology platform, we systematically compared the As binding affinity of 9 RpArsR homologs from *R. palustris* CGA009 and identified S-RpArsR2 as a high-efficiency adsorbent that significantly lowered As(III) accumulation in plant *Amaranthus tricolor*. Through the quantitative analysis the binding affinity of multiple RpArsRs and As, the complex arsenic resistance mechanism was elucidated in this study. Beyond arsenic research, this method can be broadly applied to other protein-ligand systems and provides a novel strategy for developing highly selective adsorbent materials, with broad implications for environmental bioremediation and metalloregulatory research.

## Results and discussion

### Phylogenetic analysis and sequence diversity of ArsR proteins from *R. palustris* CGA009

**FIGURE 1:**
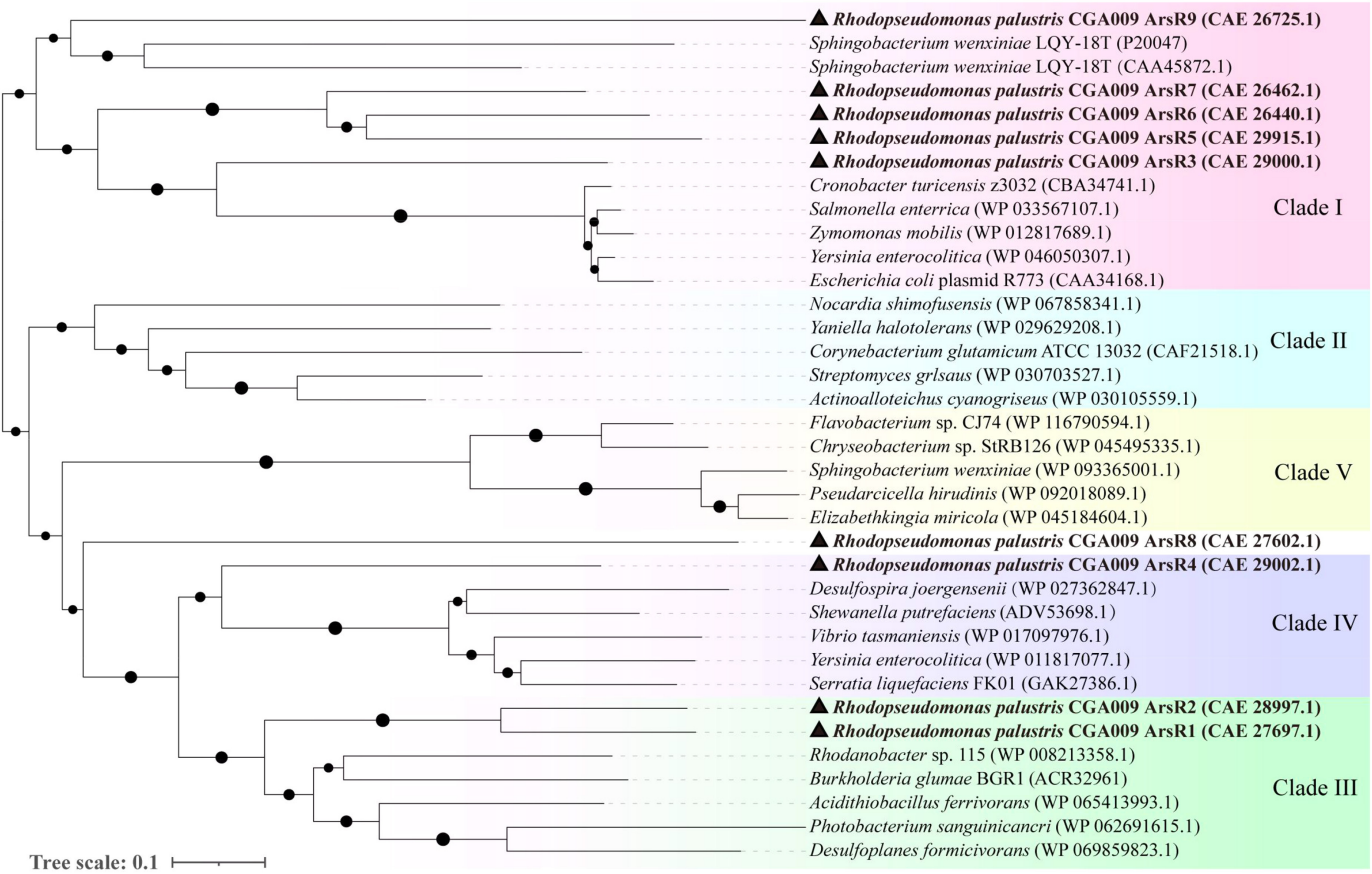
Evolutionary relationships of RpArsRs in *R. palustris* CGA009. A neighbor-joining phylogenetic tree reveals that the ArsR family clusters into five distinct clades. Nine RpArsR proteins from *R. palustris* CGA009 (accession numbers in parentheses) are highlighted and fall into clades I, III, and IV (marked with black triangles).

*R. palustris* CGA009 exhibits exceptional arsenic resistance. Genomic analysis revealed an unusually large repertoire of nine RpArsR homologs (Supplementary Table 2). To explore their evolutionary relationships, we conducted a phylogenetic analysis using ArsR homologs from *R. palustris* and 27 other bacterial strains (Chen et al., 2017), As shown in Figure 1, these ArsR proteins clustered into four evolutionarily distinct clades. RpArsR3, 5, 6, 7, and 9 grouped into Clade I (EcArsR type), RpArsR1 and RpArsR2 into Clade III (AfArsR type), RpArsR4 into Clade IV (SpArsR type), and RpArsR8 formed a separate branch closely related to Clade IV. The distribution of the nine RpArsR proteins across multiple clades suggests a complex and adaptive regulatory system evolved to cope with diverse arsenic stress conditions. Sequence analysis revealed substantial diversity in cysteine residue content among the nine RpArsRs (Supplementary Figure 1), which directly influences their ability to coordinate As(III). RpArsR1 and RpArsR2, containing five cysteine residues (Cys91–92, Cys99, Cys109, Cys110), have the highest potential to form multiple three-coordinate As(III)-binding sites. RpArsR4 has four cysteines, RpArsR3 has three, RpArsR5, 6, 7, 8 have two, and RpArsR9 has only one, making it unlikely to form a functional As(III)-binding site. This structural and functional diversity among ArsR homologs in *R. palustris* CGA009 may reflect evolutionary adaptation to complex environmental arsenic challenges, consistent with recent studies suggesting that multiple binding sites in ArsR can confer adaptive advantages (Chen et al., 2025).

### Immobilization of RpArsRs on biosilica spheres (ELP-SpyCatcher@SiO_2_, abbreviated as S)

All recombinant RpArsR proteins were successfully expressed and purified, as confirmed by SDS-PAGE analysis (Supplementary Figure 2). The observed molecular weights closely matched their theoretical values: RpArsR1 (13.2 kDa), RpArsR2 (14.1 kDa), RpArsR3 (12.7 kDa), RpArsR4 (14.5 kDa), RpArsR5 (15.7 kDa), RpArsR6 (17.3 kDa), RpArsR7 (13.7 kDa), RpArsR8 (12.8 kDa), RpArsR9 (13.3 kDa), ELP-SpyCatcher (35.0 kDa), and RFP-SpyTag (29.3 kDa). To construct solid-phase arsenic adsorbents (S-RpArsRs), biosilica spheres (S) functionalized with ELP-SpyCatcher were synthesized and optimized for uniformity. Initial attempts based on previous protocols (Lin et al., 2019), yielded aggregated particles unsuitable for quantitative assays. By optimizing the silicification process using 0.1 mM HCl, we generated monodisperse biosilica microspheres with diameters ranging from 0.54 to 1.23 μm, as confirmed by dynamic light scattering and electron microscopy. The final S suspension was adjusted to 1.00 g/L (based on SiO_2_ content), with a particle concentration estimated at 1.657 ± 0.076 × 10^11^ particles/L by microscopic counting. Using Avogadro’s constant, the concentration of S was calculated as 2.753 × 10^–7^ μM. The particles numbers of S was calculated as 1.657 ± 0.076 × 10^11^/g, the molar mass of S was 3.632 × 10^12^g/mol. This optimized platform allowed for precise stoichiometric control of S-RpArsR, enabling robust evaluation of arsenic-binding affinities.

### Synthesis and characterization of solid-phase arsenic adsorbents (S-RpArsR)

**FIGURE 2:**
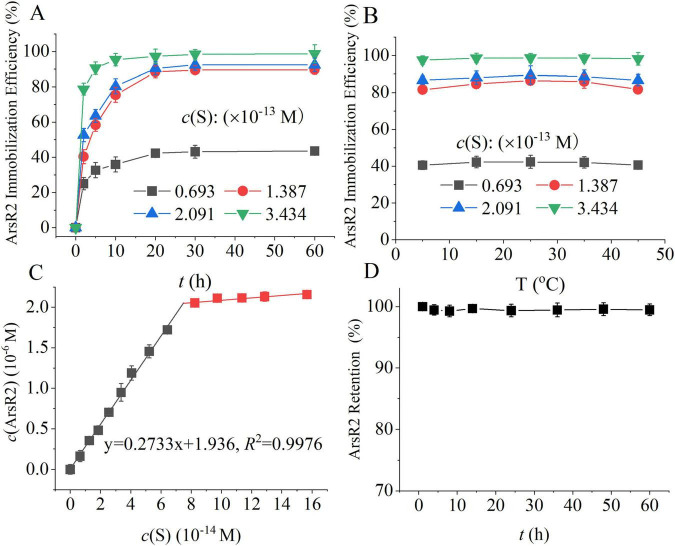
Synthesis and characterization of solid-phase arsenic absorbents (S-RpArsR2). **(A)** Effect of incubation time and biosilica sphere concentration on RpArsR2 immobilization. **(B)** Effect of temperature on RpArsR2 immobilization. **(C)** Quantification of RpArsR2 fixation for 20 min incubation at 25°C. **(D)** Stability assessment of the RpArsR2 synthesis. The concentration of the RpArsR2-SpyTag construct [*c*(RpArsR2-SpyTag)] was 2.157 × 10^– 6^M. Solid-phase arsenic absorbents (S-RpArsR2) were prepared as described in Materials and methods. Data are mean ± SD (*n* = 3 biological replicates). Statistical significance determined by one-way ANOVA with LSD *post-hoc* test (*P* < 0.05).

S-RpArsR were synthesized by covalently immobilizing RpArsR/RFP-SpyTag fusion proteins onto S via the specific and spontaneous SpyCatcher/SpyTag reaction. As an initial validation, S-RFP particles were prepared as a fluorescent control and confirmed excellent particle dispersion via confocal microscopy (Supplementary Figure 3), demonstrating the platform’s suitability for quantitative arsenic-binding studies. Based on our previous findings showing that RpArsR2 confers strong resistance to As(III), we selected this variant as the model protein for arsenic-binding studies. We systematically investigated the effects of incubation time, S concentration, and temperature on the synthesis of S-RpArsR (Figure 2). The amount of immobilized RpArsR2 increased with both incubation time and S concentration (0.693–3.434 × 10^–7^ μM), reaching a maximum at 20 min and 25°C (Figure 2A). Notably, temperature variations between 4 and 45°C had minimal impact on RpArsR2 immobilization efficiency (Figure 2B), consistent with the spontaneous SpyTag/SpyCatcher reaction kinetics (Tian et al., 2021). Under optimized conditions (25°C, 20 min), a strong linear correlation (*R*^2^ = 0.9976) was observed between S concentration and RpArsR2 loading, with a maximum immobilization capacity of (2.733 ± 0.064) × 10^7^ molecules per biosilica sphere (Figure 2C). The resulting S-RpArsR2 conjugates demonstrated excellent stability, showing less than 4% protein release after 60 h at 25°C (Figure 2D). This minor leakage was likely due to non-specific adsorption rather than disruption of covalent SpyCatcher/SpyTag linkages. Overall, this robust platform enables precise assessment of arsenic-binding affinities and holds promise for development of advanced bioadsorbent materials for environmental remediation.

### Morphology and structure of S-RpArsR

**FIGURE 3:**
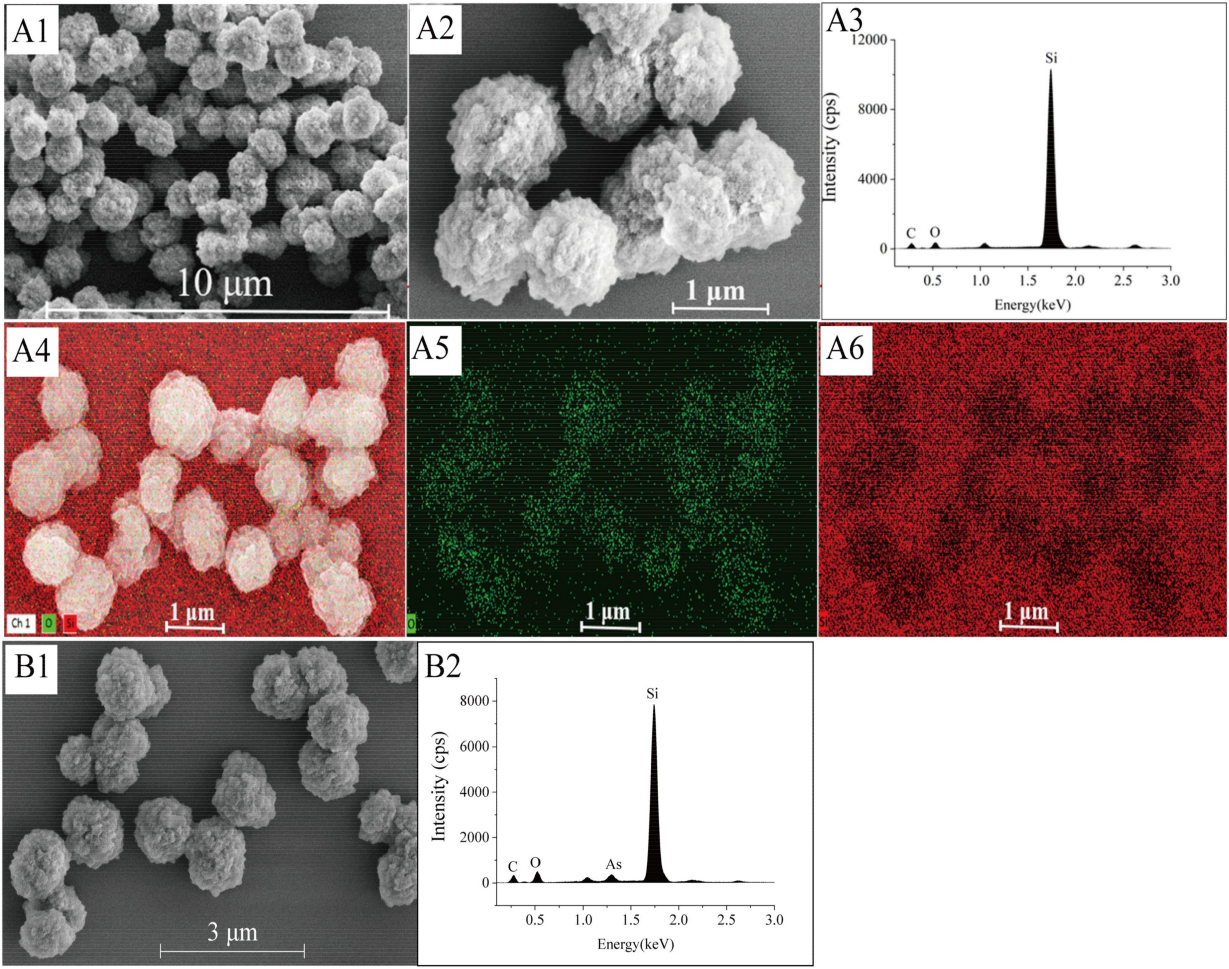
Structural and elemental characterization of S-RpArsR2 before and after As(III) adsorption. The structure of the synthesized S-RpArsR2 particles was confirmed through morphological analysis using field emission scanning electron microscopy (FE-SEM). The expected elemental composition of the biosilica matrix S-RpArsR—primarily silicon and oxygen, with carbon accounting for remaining structure from organic components—is effectively confirmed using Energy-Dispersive X-ray Spectroscopy (EDS). **(A1,A2)** FE-SEM images showing the morphological features of S-RpArsR2 before As(III) adsorption (at 10 and 1 μm scales, respectively). **(A3)** EDS spectrum before adsorption, highlighting the elemental composition dominated by silicon and oxygen. EDS-Mapping before adsorption for **(A4)** overlay of Si (in red) and O (in green), **(A5)** Isolated oxygen mapping and **(A6)** Isolated silicon mapping. **(B1)** FE-SEM image after adsorption, showing morphological changes after-As(III) exposure (scale: 3 μm). **(B2)** EDS spectrum after adsorption, now indicating the presence of arsenic along with Si and O.

Field emission scanning electron microscopy (FE-SEM) revealed that S-RpArsR2 particles exhibited uniform spherical morphology with a size range of 0.54–1.23 μm (average: 0.81 μm), which is slightly larger than previously reported biosilica spheres (0.2–0.6 μm) (Cai et al., 2020). The particles also featured rough surfaces with granular protrusions (Figures 3A1,A2). Energy-dispersive X-ray spectroscopy (EDS) confirmed the expected elemental composition (Si, O, and C) of the biosilica matrix (Figure 3A3), consistent with earlier findings (Lin et al., 2019). EDS mapping further demonstrated homogeneous distribution of Si and O across the particle surface (Figures 3A4–A6), indicating successful functionalization with RpArsR2. In the presence of As(III), particle morphology remained largely unchanged (Figure 3B1), but EDS spectra clearly showed the appearance of arsenic signals, confirming successful As(III) adsorption (Figure 3B2).

### Adsorption kinetics of S-RpArsR2 for As(III)

Both S and S-RpArsR2 exhibited the capacity to adsorb As(III) over a wide pH range (3.0–11.0), with optimal performance observed under neutral to mildly acidic or alkaline conditions (pH 6.0–8.0) (Supplementary Figure 4A). S-RpArsR2 showed significantly higher adsorption capacity than S alone, maintaining over 60% of its maximum adsorption efficiency even at extreme pH values (3.0 or 11.0). Kinetic analysis revealed rapid As(III) uptake by S-RpArsR2, with most adsorption occurring within the first 4 h and equilibrium reached by 12 h (Supplementary Figure 4B). To investigate the adsorption kinetics, the data from Supplementary Figure 4B were fitted to two commonly used models: the pseudo-first-order kinetic model [ln(Q_e_–Q_t_) = lnQ_e_ – k⋅t] and the pseudo-second-order model [t/Q_t_ = 1/(k⋅Q_e_^2^) + t/Q_e_], where Q_e_ is the equilibrium adsorption amount, Q_t_ is the amount adsorbed at time t, and k is the rate constant (Aguado et al., 2009). Linearized forms of both models were plotted as ln(Q_e_–Q_t_) versus t (Supplementary Figure 4C) and t/Q_t_ versus t (Supplementary Figure 4D). The corresponding linear fits and regression coefficients (R^2^ values) are summarized in Table 1. The adsorption behavior of S-RpArsR2 was best described by the pseudo-second-order model (R^2^ > 0.996), with the experimental Q_e_ values closely matching theoretical predictions. In contrast, the pseudo-first-order model showed poor fitting and lower accuracy in Q_e_ estimation. These results indicate that S-RpArsR2 significantly enhances both the rate and capacity of As(III) adsorption. At a concentration of 1.00g/L, S-RpArsR2 completely removed 100μg/L As(III) from solution, while S alone removed less than 60%, highlighting the effectiveness of RpArsR-functionalized biosilica in arsenic adsorption.

**TABLE 1:** Kinetic parameters of As(III) adsorption.

Kinetic model	Adsorbents	ln(Q_*e*_-Q_t_) = lnQ_e_ – k⋅t	*k* (h^–1^)	Q_e_ (μg/g)	*R* ^2^	Q_c_ (μg/g)
I	S	Ln(Q_*e*_-Q_t_) = 3.66–0.174 t	0.174 ± 0.022	38.6	0.9210	58.1
	S-RpArsR2	Ln(Q_*e*_-Q_t_) = 4.38–0.254 t	0.254 ± 0.020	79.8	0.9688	101.74
	Adsorbents	t/Q_t_ = 1/(k⋅Q_e_^2^) + t/Q_e_	*k* (g/(μg⋅h))	Q_e_ (μg/g)	*R* ^2^	Q_c_ (μg/g)
II	S	t/Q_t_ = 0.01651 + 0.0237 t	0.0165 ± 0.0025	60.6	0.9968	58.1
	S_2_-RpArsR2	t/Q_t_ = 0.00928 + 0.0135 t	0.0665 ± 0.00076	107.8	0.9989	101.74

I, pseudo-first-order [ln(Q_*e*_-Q_t_) = lnQ_*e*_-k⋅t]. II, pseudo-second-order [t/Q_t_ = 1/(k⋅Q_e_^2^) + t/Q_*e*_].

### Thermodynamic analysis of As(III) adsorption by S-RpArsR

**FIGURE 4:**
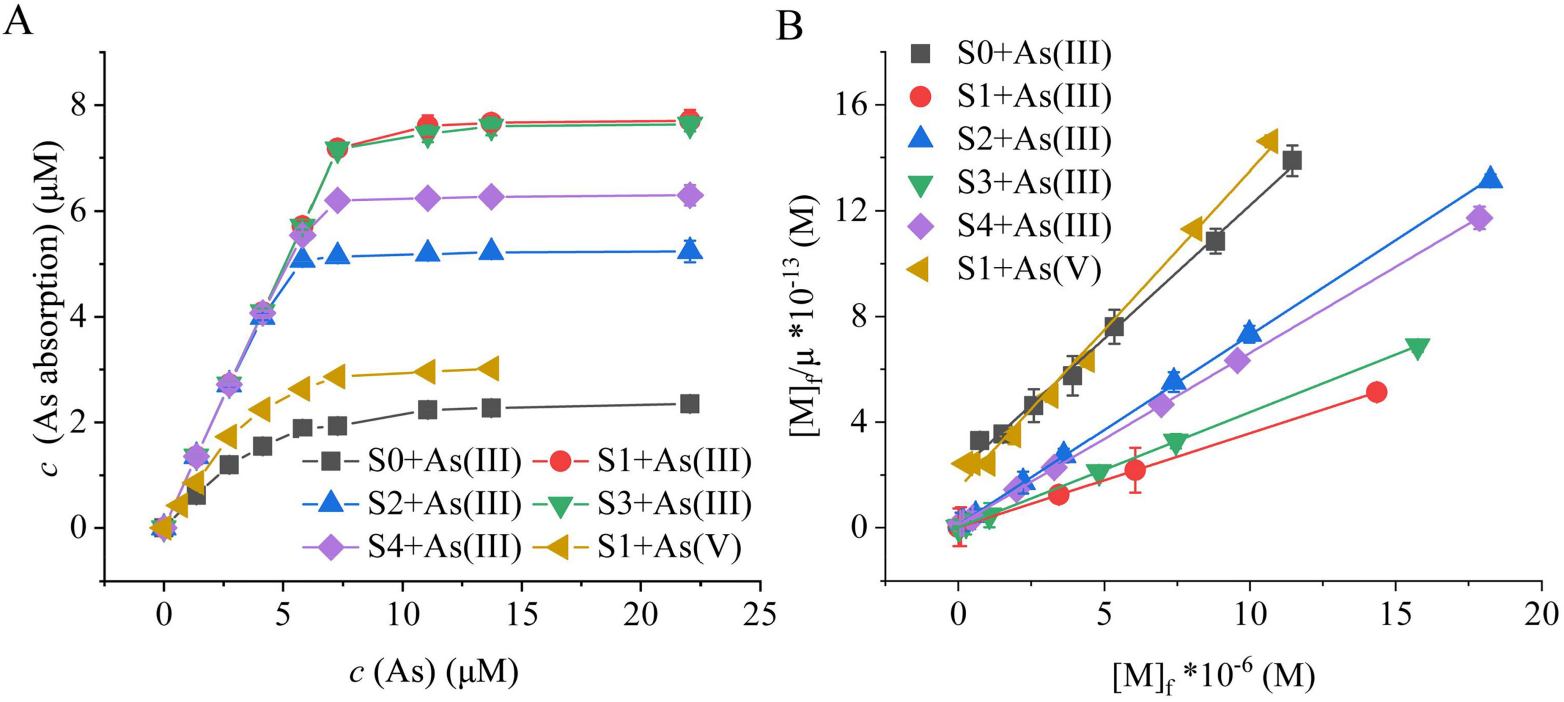
Thermodynamic analysis of As(III) adsorption by S-RpArsR2 variants. Thermodynamic analysis of As(III) adsorption capacity of S-RpArsR2 variants was assayed as described in Materials and methods. **(A)** Adsorption isotherm curves depicting As(III) uptake by different S-RpArsR2 preparations. **(B)** Scatchard plot analysis to assess the binding affinity and the number of adsorption sites, evaluating homogeneity versus heterogeneity of binding. S0: Control with only biosilica spheres (2.753 × 10^– 7^μM). S1 and S2: Purified S-RpArsR2 variants at differing loading concentrations (2.733 × 10^7^ and 1.811 × 10^7^ mol/mol, respectively). S3 and S4: S-RpArsR2 preparations in which the protein was immobilized directly from crude lysate: performed once in the case of S3, and twice for S4. Data are mean ± SD (*n* = 3 biological replicates). Statistical significance determined by one-way ANOVA with LSD *post-hoc* test (*P* < 0.05)


To evaluate the As(III) adsorption performance of S-RpArsR2, four variants (S1–S4) with different RpArsR2 immobilization densities were prepared. All S-RpArsR2 variants exhibited significantly higher As(III) adsorption than the control biosilica spheres (S0), with S1 and S3 showing the highest uptake, correlating positively with protein loading (Figure 4A). Scatchard analysis was performed under the assumption that the As-binding sites on S–RpArsR2 are independent and identical, with each site exhibiting the same binding capacity for As(III). Under equilibrium conditions, the binding behavior can be described by the following equations (1) and (2), Where, M and S represent As and S-RpArsR2, respectively. *K*_*A*_ and *K*_*D*_ represent affinity constant and dissociation constant, respectively. [M]_*b*_ and [M]_*f*_ represent the concentrations of As-binding and As-free, respectively. [S] and n represent the concentrations and As adsorption site numbers of S-RpArsR2, respectively. [S]_*t*_, [S]_*b*_, and [S]_*f*_ represent the total concentrations, As-bound and As–free of S-RpArsR2, respectively. Subscript b and f mean the binding and the free. Due to [S]_*t*_ = n[S], [S]_*b*_ = [M]_*b*_, therefore, [S]_*f*_ = [S]_*t*_-[S]_*b*_ = n[S]-[M]_*b.*_ Due to μ = [M]_*b*_/[S]_*t*_, transformed equation (2) to obtain Scatchard equation (3).

$$M+S\overset{K_{A}}{⟷}M\,S$$ (1)

$$K_{A}=\frac{1}{K_{D}}=\frac{{\left[M\right]}_{b}}{{\left[M\right]}_{f}\,{\left[S\right]}_{f}}=\frac{{\left[M\right]}_{b}}{{\left[M\right]}_{f}\,\left(n\,\left[S\right]-{\left[M\right]}_{b}\right)}$$ (2)

$${\left[M\right]}_{f}/\mu =K_{D}/n+{\left[M\right]}_{f}/n$$ (3)

Take the data in Figure 4A to plot [M]_*f*_/μ vs. [M]_*f*_ according to equation (3), yielding the results shown in Figure 4B. Subsequently, selecting the linear segment in Figure 4B to perform linear fitting and yielded high correlation (*R*^2^ > 0.99), indicating good model fit. The excellent fit to pseudo-second-order kinetic model suggests that chemisorption is the rate-limiting step in the adsorption process, which is mechanistically consistent with the specific coordination binding between ArsR protein and As(III) via the thiol groups (-SH) of conserved cysteine residues in immobilized RpArsR2. The affinity constants (*K*_*A*_) and binding site numbers (n) were derived from slope and intercept values (Table 2). S-RpArsR2 variants (S1–S4) showed over 10-fold higher *K*_*A*_ than S0, with a maximum *K*_*A*_ of 3.05 × 10^7^ M. The n values (1.03–1.05) confirmed a 1:1 stoichiometry between As(III) and RpArsR2, and scaled with protein loading. Compared to As(III), As(V) exhibited markedly lower adsorption and affinity, confirming the high selectivity of S-RpArsR2. Thermodynamic analysis revealed: (1) As(III) adsorption capacity is protein-density dependent; (2) binding affinity remains consistent across variants (RSD = 4.13%); and (3) the practicality of the purification-free immobilization strategy was demonstrated, as repeated immobilization (S4) increased protein loading and enhanced As(III) adsorption capacity by 18.2% compared to single-step deposition (S3), despite a modest 7.5% reduction in *K*_*A*_, likely due to competition from impurities. It confirmed that this biomimetic approach allows accurate thermodynamic evaluation without the need for protein purification. This method provides a technically robust and efficient platform for investigating arsenic-protein interactions with high precision and reproducibility.

**TABLE 2:** The affinity constant (*K*_A_) and binding site numbers (n) of S-RpArsR2.

Adsorbents	Q (× 10^7^ mol/mol)	As	Scatchard equation	*R* ^2^	n (× 10^7^)	*K*_A_ (× 10^6^ M)
S0	0	As (III)	y = 0.9981x + 2.1491	0.9966	1.00 ± 0.024	0.477 ± 0.013
S1	2.733	As (III)	y = 0.3567x + 0.0117	0.9979	2.80 ± 0.025	30.49 ± 0.063
S2	1.811	As (III)	y = 0.5250x + 0.0186	0.9998	1.91 ± 0.024	28.22 ± 0.013
S3	once	As (III)	y = 0.4363x + 0.0141	0.9993	2.29 ± 0.025	30.94 ± 0.052
S4	twice	As (III)	y = 0.3600x + 0.0123	0.9995	2.78 ± 0.024	29.27 ± 0.107
S1	2.733	As (V)	y = 1.2027x + 1.4811	0.9929	0.83 ± 0.024	0.812 ± 0.042

S0: Control with only biosilica spheres (2.753 × 10^–7^ μM). S1 and S2: Purified S-RpArsR2 variants at differing loading concentrations (2.733 × 10^7^ and 1.811 × 10^7^ mol/mol, respectively). S3 and S4: S-RpArsR2 preparations in which the protein was immobilized directly from crude lysate: performed once in the case of S3, and twice for S4.

### Assessment of immobilization effects on protein-As(III) affinity

To assess whether immobilization affects the arsenic-binding activity of S-RpArsR2, we conducted parallel competitive adsorption experiments comparing (1) immobilized RpArsR2 (S-RpArsR2, denoted as R) versus free RpArsR2, and (2) S-RpArsR2 (R) versus BSA (P) as a non-specific control protein. In this competitive system, both proteins (R and P) compete for As(III), and at equilibrium, the interaction is described by a series of equations (Equations 4–8). Here, subscripts t, f, and b refer to total, free, and bound concentrations, respectively. Specifically, these relationships are expressed as [R]_*f*_ = [R]_*t*_ − [R]_*b*_, [R]_*t*_ = n[S], [P]_*b*_ = [As (III)]_*T*_ − [R]_*b*_ − [As]_*f*_, and [P]_*f*_ = [P]_*t*_ − [P]_*b*_. Here, [R], [P], [S] and [As (III)] represent the concentrations of R, P, S-RpArsR2 and As(III), respectively. Due to the high affinity of R and As(III), [As]_*f*_ was disregarded when As(III) concentration is not excess.


$$R+A\,s\,\left(I\,I\,I\right)\underset{K_{\mathrm{D1}}}{⟷}R-A\,s\,\left(I\,I\,I\right)$$ (4)

$$P+A\,s\,\left(I\,I\,I\right)\underset{K_{\mathrm{D2}}}{⟷}P-A\,s\,\left(I\,I\,I\right)$$ (5)

$$\frac{1}{K_{\mathrm{D1}}}=\frac{{\left[R\right]}_{\text{b}}}{{\left[R\right]}_{\text{f}}]{\left[AsIII\right]}_{\text{f}}]}$$ (6)

$$\frac{1}{K_{\mathrm{D2}}}=\frac{{\left[R\right]}_{\text{b}}}{{\left[R\right]}_{\text{f}}[As{\left(III\right)}_{\text{f}}}$$ (7)

$$\frac{{\left[R\right]}_{\text{f}}}{{\left[R\right]}_{\text{b}}}=\frac{{\left[P\right]}_{\text{f}}}{{\left[P\right]}_{\text{b}}}•\frac{K_{\mathrm{D1}}}{K_{\mathrm{D2}}}$$ (8)

**FIGURE 5:**
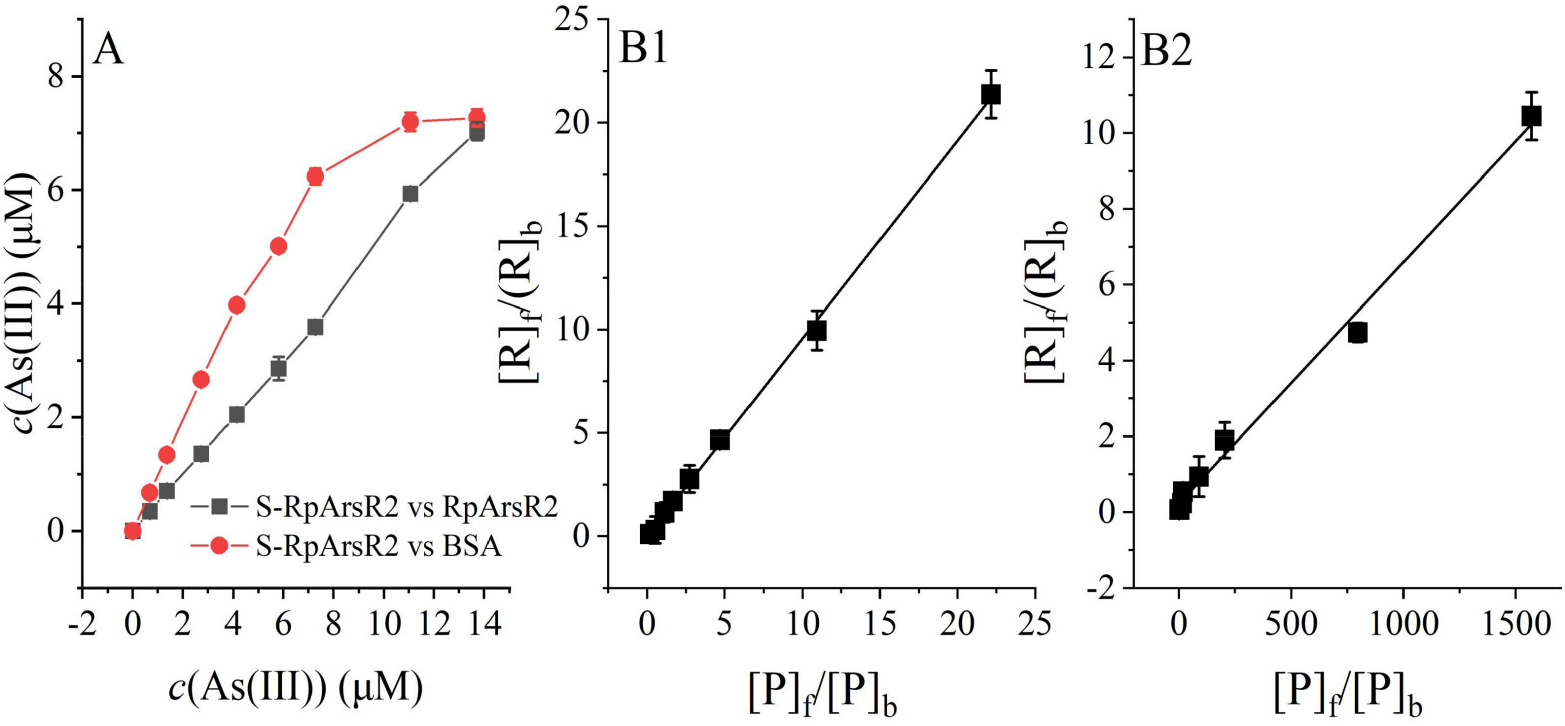
Assessment of immobilization effects on S-RpArsR2-As(III) Affinity. The potential immobilization-induced effect in arsenic-binding activity of S-RpArsR2 was evaluated, as described in Materials and methods. Binding interactions were assessed via competition assays between immobilized purified protein (S-RpArsR2) and free purified protein (either RpArsR2 or BSA). **(A)** Adsorption isotherms comparing As(III) binding by S-RpArsR2 (gray squares) versus free protein samples (red circles). **(B1)** Scatchard-style plots of [R]_f_/[R]_b_ versus vs. [P]_f_/[P]_b_ for S-RpArsR2. **(B2)** Scatchard-style plots of [R]f/[R]b versus vs. [P]f/[P]b for BSA, as a negative control to confirm specificity and validate the analytic method. Data are mean ± SD (*n* = 3 biological replicates). Statistical significance determined by one-way ANOVA with LSD *post-hoc* test (*P* < 0.05).

Assuming a 1:1 stoichiometry and negligible free As(III) due to high binding affinity, we plotted [R]_*f*_/[R]_*b*_ vs. [P]_*f*_/([P]_*b*_) results in Figure 5B1, using data from Figure 5A. Linear fitting of the data (Figure 5B1) allowed us to calculate the ratio of *K*_*D*1_/*K*_*D*2_, from which *K*_*A*_ value of free protein was further derived. The calculated *K*_*A*_ of free RpArsR2 was 3.128 × 10^7^ M, closely matching the value for immobilized S-RpArsR2 (3.049 × 10^7^ M) (Table 2 and Supplementary Table 1), with a deviation of less than 3%. This consistency indicates that immobilization did not alter the As(III)-binding affinity of RpArsR2. To further validate the competitive method, we tested BSA as a non-specific control. The resulting *K*_*A*_ value (1.80 × 10^5^ M) (Figure 5B2 and Supplementary Table 3) was in excellent agreement with that obtained via independent fluorescence quenching (1.39 × 10^5^ M, *n* = 1.07) (Supplementary Figure 5 and Supplementary Table 4), supporting the reliability and accuracy of our method. As(III) specifically coordinates with cysteine thiol (-SH) groups of protein, forming stable complexes. We employed a covalent SpyCatcher/SpyTag self-assembly strategy to immobilize RpArsRs onto biosilica spheres, ensuring uniform orientation while maintaining the accessibility of the metal-binding domain. This immobilization process preserved the cysteine-mediated coordination and did not alter binding affinity. Because the SpyTag/SpyCatcher covalent interaction is highly specific and orthogonal, the structural and functional preservation observed for RpArsR2 is expected to be representative of other homologs. By rigorously validating this principle with RpArsR2, we established a reliable proof-of-concept that enables high-throughput, purification-free screening of the remaining eight RpArsR homologs, without the need for redundant protein purification steps.

### Arsenic-binding affinities of nine RpArsRs from *R. palustris* CGA009

**FIGURE 6:**
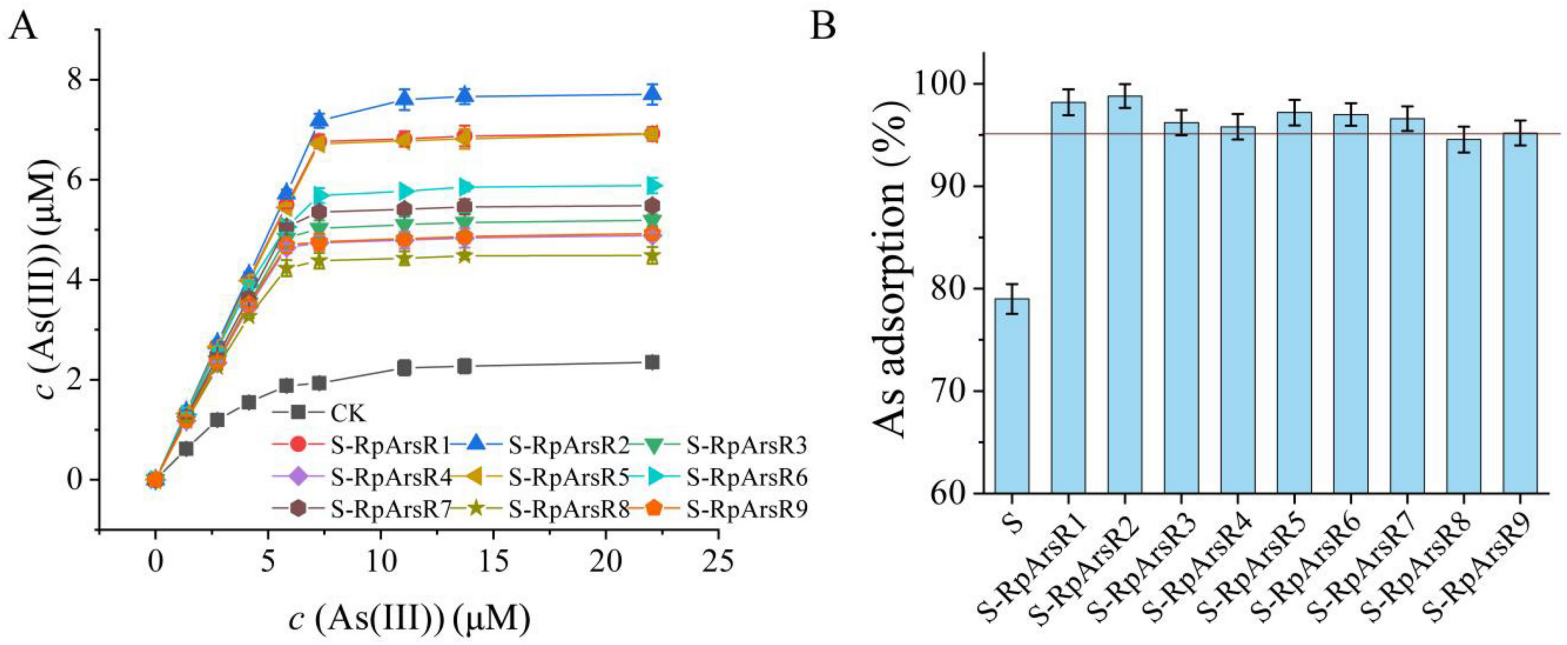
Comparison of As(III) binding affinity across RpArsR homologs and the impact of coexisting heavy metals. **(A)**. Adsorption isotherms for nine S-RpArsR complexes (colored curves), only biosilica spheres was used as a control (CK). **(B)** Impact of heavy metal ions on As(III) adsorption efficiency. Each S-RpArsR variant (1.00g/L or 2.753 × 10^– 7^ μM) and biosilica spheres only (S, control) was exposed to 13.3μM As(III) both with and without coexisting metal ions (13.3μM Cu^2+^, Zn^2+^, Pb^2+^, Co^2+^, CrO_7_^2–^, Ni^2+^) in pH7.0 TB74s buffer. The impact of various heavy metal ions was shown in y-axis, which represents the ratio of As adsorption in the presence of heavy metals to that in metal-free controls (normalized as 100%). It reflects the percentage of retained binding capacity under interference conditions. A red horizontal line marks the 95% retention threshold. As(III) adsorption of nine S-RpArsR complexes and S were quantitatively determined using biomimetic self-assembly approach, as described in Materials and methods. Data are mean ± SD (*n* = 3 biological replicates). Statistical significance determined by one-way ANOVA with LSD *post-hoc* test (*P* < 0.05).

*R. palustris* CGA009 exhibits high As-resistance, which is attributed to the presence of at least three *ars* operons (Zhao et al., 2015; Ke et al., 2018), and the presence of nine phylogenetically diverse ArsR homologs clustered into four clades. To investigate their functional divergence, the As(III)-binding affinities of all nine RpArsRs were quantitatively assessed using the biomimetic self-assembly approach (Figure 6A). All S-RpArsR conjugates exhibited significantly enhanced As(III) adsorption compared to biosilica controls (*P* < 0.05), with slope increases in the linear adsorption regions corresponding to 4.2–18.7-fold affinity enhancements. However, their adsorption capacities varied, with RpArsR2 showing the highest As(III) uptake.

Scatchard analysis of the isotherms (Supplementary Figure 6) revealed strong linear correlations (*R*^2^ > 0.996), allowing for the accurate determination of the affinity constant (*K*_*A*_) and binding site density (n) for each variant (Table 3). Except for RpArsR2, which reached saturation loading, all other RpArsRs showed lower site occupancy (*n* = 1.671 – 2.527 × 10^7^ sites/sphere), suggesting incomplete surface coverage. *K*_*A*_ values varied over an order of magnitude, ranging from 2.829 × 10^6^ to 3.049 × 10^7^ M, following the order: RpArsR2 > RpArsR1 > RpArsR5 > RpArsR6 > RpArsR7 > RpArsR3 > RpArsR9 > RpArsR4 > RpArsR8. This trend aligns with their phylogenetic classification and highlights functional differentiation. Overall, the results demonstrate that all nine RpArsRs from *R. palustris* CGA009 bind As(III), with several exhibiting high binding affinities that likely contribute to intracellular arsenic sequestration and reduced toxicity by limiting As(III) availability to other cellular targets. Previous studies have shown that heterologous expression of ArsRs in engineered bacteria enhances both arsenic accumulation and tolerance (Ke et al., 2018; Maleki and Shahpiri, 2022; Ghahghaei and Shahpiri, 2025). In bacteria harboring multiple ArsR homologs, these regulators may compete for As(III) substrates, potentially forming complex interaction networks that affect gene regulation and arsenic metabolism. In *R. palustris* CGA009, such interactions among multiple RpArsRs may contribute to a finely tuned regulatory system, although this remains to be experimentally validated. Interestingly, although the *ars* operon of CGA009 contains *arsM* [regulated by RpArsR4 and typically induced by As(III)], no arsenic methylation products were detected. This absence may result from efficient arsenic efflux mechanisms reducing intracellular As(III) levels, and/or competitive binding by highly expressed RpArsRs-particularly RpArsR2, which exhibits the highest As(III) affinity (Table 3). RpArsR2, which contains five cysteine residues, exhibited the highest *K*_*A*_ value, indicating a strong arsenic binding capacity. The combination of its binding affinity and expression level may influence intracellular As(III) availability and potentially affect downstream processes such as ArsM-mediated methylation; however, this regulatory effect remains speculative and requires further experimental validation. RpArsR3 (with three cysteines) and RpArsR9 (with only one cysteine) showed comparable *K*_*A*_ values, suggesting that residues other than cysteine may contribute to As(III) binding. Notably, RpArsR5, RpArsR6, and RpArsR7, despite having similar cysteine counts, displayed markedly different *K*_*A*_ values (Table 3), implying that the binding affinity is also influenced by structural context and specific residue arrangements. These findings suggest that ArsR-regulated arsenic resistance extends beyond canonical *ars* operons and involves broader, non-redundant protective roles-contributing to the exceptional arsenic resistance observed in CGA009.

**TABLE 3:** The affinity constant (*K*_A_) and binding site numbers (n) of nine S-RpArsR complexes toward As(III).

Adsorbent	Scatchard equation	*K*_A_ × 10^6^(M)	n (× 10^7^)	*R* ^2^
S	y = 0.9981x + 2.1491	0.477 ± 0.013	1.002 ± 0.024	0.9966
S-RpArsR1	y = 0.3961 x + 0.0306	12.987 ± 0.061	2.525 ± 0.072	0.9996
S-RpArsR2	y = 0.3567x + 0.0117	30.487 ± 0.063	2.803 ± 0.025	0.9979
S-RpArsR3	y = 0.5201 x + 0.1447	3.594 ± 0.042	1.923 ± 0.057	0.9995
SRp-ArsR4	y = 0.5511 x + 0.1732	3.182 ± 0.068	1.815 ± 0.033	0.9992
S-RpArsR5	y = 0.3958 x + 0.0428	9.248 ± 0.046	2.527 ± 0.094	0.9995
S-RpArsR6	y = 0.4634 x + 0.0675	6.865 ± 0.052	2.158 ± 0.043	0.9989
S-RpArsR7	y = 0.4937 x + 0.1113	4.436 ± 0.055	2.026 ± 0.038	0.9996
S-RpArsR8	y = 0.5984 x + 0.2000	2.829 ± 0.024	1.671 ± 0.013	0.9988
S-RpArsR9	y = 0.5477 x + 0.1639	3.342 ± 0.042	1.826 ± 0.029	0.9994

### Influence of heavy metal ions on As(III) Adsorption by S-RpArsRs

To evaluate the binding specificity of the S-RpArsR2 conjugates toward As(III), we examined its performance in the presence of six common heavy metals [Cu(II), Zn(II), Pb(II), Cd(II), Cr(VI), and Ni(II)], each at 10-fold excess (13.3 μM) relative to As(III) (1.33 μM). The results revealed clear differences between the unmodified biosilica (S) and the S-RpArsR variants. As shown in Figure 6B, the control S particles exhibited a significant 21.3 ± 2.1% decrease in As(III) adsorption under metal interference (*P* < 0.05, *n* = 3). In contrast, eight of the nine S-RpArsR conjugates retained over 95% of their original As(III) adsorption capacity, indicating strong selectivity. Only S-RpArsR8 showed a slightly greater reduction (5.5%), which aligns with its lower binding affinity (*K*_*A*_ = 2.829 × 10^6^ M). The red horizontal line in Figure 6B denotes the 95% retention threshold, indicating minimal interference by heavier metal ions. Notably, S-RpArsR1 and S-RpArsR2 demonstrated exceptional selectivity and stability, maintaining high As(III) uptake despite heavy metal competition. These findings underscore the robustness of ArsR-functionalized adsorbents, offering a clear advantage for selective arsenic removal from multi-metal contaminated water sources.

### Effect of S-RpArsR application on plant growth

ArsR proteins contain cysteine thiol groups that enable coordination with arsenicals, making them promising candidates for arsenic bioremediation. Previous studies demonstrated that the overexpression of *E. coli* ArsR enhances intracellular arsenic accumulation (Zhao et al., 2015). To further evaluate the physiological impact of S-RpArsR mediated arsenic adsorption, we selected three S-RpArsR variants: S-RpArsR2, S-RpArsR3, and S-RpArsR8, which showed a different As(III) binding affinities, for hydroponic assays with *Amaranthus tricolor*. After 4-day exposure to 2.67 μM As(III), plants in the negative control group (CK-N) remained healthy, while those in the positive control (CK-P) and biosilica-only group (S) showed visible wilting. In contrast, plants treated with the three S-RpArsR variants exhibited healthy growth comparable to that of the CK-N group (Supplementary Figure 7), indicating mitigation of arsenic toxicity. After exposure to 200 μg/L As(III) for 4 days, as shown in Table 4, S-RpArsR treatments significantly increased plant water content (*P* < 0.05) and reduced shoot As(III) accumulation in the following order: S-RpArsR2 > S-RpArsR3 > S-RpArsR8 > S. Notably, S-RpArsR2 (the highest-affinity binder) resulted in the lowest shoot arsenic accumulation (42.7 ± 1.79 μg/g). A strong correlation (R^2^ = 0.94) was observed between *in vitro* As(III) binding affinities and *in planta* mitigation efficiency.

**TABLE 4:** Effects of S-RpArsRs treatments on plant *Amaranthus* tricolor.

Treatments	As(III) (μM)	Weight (g) Mean ± SD	Water contents (%) Mean ± SD	As contents (μg/g) Mean ± SD
CK-N	0	5.15 ± 0.14^a^	90.0 ± 0.23^a^	0.1 ± 0.11^a^
CK-P	2.67	2.44 ± 0.11^bc^	82.3 ± 3.11^b^	78.5 ± 1.02^b^
S (Biosilica-only)	2.67	2.47 ± 0.33^c^	85.1 ± 3.42^bc^	65.0 ± 0.22^c^
S-RpArsR2	2.67	5.62 ± 1.08^a^	88.4 ± 1.01^ac^	42.7 ± 1.79^d^
S-RpArsR3	2.67	5.03 ± 1.76^a^	87.8 ± 1.21^ac^	49.3 ± 0.26^e^
S-RpArsR8	2.67	4.23 ± 0.47^a^	87.2 ± 0.23^ac^	52.5 ± 1.03^f^

Key physiological metrics, including plant biomass (weight), water content (hydration status), and tissue As(III) concentration, were measured under different treatments as described in Materials and methods. CK-N represents the negative control (no As(III) stress); CK-P represents the positive control (2.67 μM As(III) stress only); S represents the biosilica-only control [biosilica spheres + 2.67 μM As(III)]. Data are mean ± SD (*n* = 3 biological replicates). Statistical significance determined by one-way ANOVA with LSD *post-hoc* test. Different letters indicate significant differences (*P* < 0.05).

For arsenic removal, the adsorption-based methods, including physical, chemical, and biological strategies, are widely used to control arsenic pollution, most adsorbents are optimized for As(V) removal. Effective As(III) removal typically requires prior oxidation to As(V), increasing operational complexity. Their limited binding capacity and biosafety concerns hinder practical application. Although engineered bacteria and synthetic biology approaches based on *ars* operons have been widely explored for As(III) bioremediation (Hui et al., 2024), their practical application is often constrained by limited binding capacity and biosafety concerns. In this study, the biosilica-immobilized protein adsorbent S-RpArsR2 demonstrated the highest As(III) binding affinity among the nine RpArsRs tested. Hydroponic experiments using *Amaranthus tricolor* revealed that S-RpArsR variants, particularly S-RpArsR2, significantly alleviated arsenic-induced phytotoxicity and reduced As accumulation in plant tissues. These findings underscore the practical potential of high-affinity S-RpArsRs, as effective and environmentally friendly material for arsenic removal and mitigation of arsenic stress in plants (Supplementary Figure 8). Furthermore, this strategy of combining biomimetic materials (biosilica) with specific microbial functional components (RpArsR) to protect plants aligns with the emerging paradigm of synergistic nanobioremediation. As recently reported, integrating nanomaterials with biological systems offers a highly promising and sustainable approach for enhancing heavy metal detoxification and mitigating plant stress (Hussain and Kabir, 2026). Our S–RpArsR platform, applied in a purification-free manner after initial validation, provides a novel, bio-inspired, and scalable addition to such synergistic remediation technologies.

## Limitations and future directions

We examined the performance of S-RpArsRs in the presence of six common heavy metals, S-RpArsR1 and S-RpArsR2 demonstrated exceptional selectivity and stability, maintaining high As(III) uptake under competitive conditions. However, the complexity of natural soils and wastewaters, particularly the presence of organic matter, may influence practical application performance. Structurally, solid-phase immobilization also presents inherent challenges for broader application. Surface anchoring of proteins may induce steric crowding, restrict conformational flexibility, and potentially disrupt higher-order structural assembly of larger multimeric proteins, thereby affecting their activity. Future work should focus on optimizing this platform through protein engineering approaches, including the design of flexible linkers to alleviate steric hindrance and restore structural dynamics. Furthermore, this biomimetic approach can be extended to other environmental contaminants by employing alternative specific regulators or enzymes. Ultimately, field-scale validation will be essential to facilitate translation of this technology into scalable nanobioremediation applications.

## Conclusion

In this study, a novel solid-phase arsenic adsorbent (S-ArsR) was synthesized based on SpyCatcher/SpyTag specific recognition, and a novel biomimetic self-assembly platform for the quantitative evaluation of ArsR–arsenic interactions was established. Through this approach, nine RpArsR homologs from *R. palustris* CGA009 were systematically analyzed, revealing significant variations in As(III)-binding affinities. Our quantitative analysis reveals functional divergence among ArsR homologs. While some high-affinity variants (e.g., RpArsR2) contain higher cysteine content and exhibit exceptionally strong As(III) binding (*K*_A_ > 10^7^ M), other ArsRs with comparable cysteine content display different binding affinities. These results indicate that binding affinity is determined by a combination of cysteine content and structural context. This diversity suggests a complex, non-redundant detoxification network that likely contributes to the extreme arsenic resistance of *R. palustris* CGA009. As a highly selective solid-phase bioadsorbent, S–RpArsR2 remains stable under heavy metal interference and significantly reduces arsenic phytotoxicity in *Amaranthus tricolor*, validating its potential for environmental applications. This immobilization strategy, applied in a purification-free manner after initial validation, enables precise, scalable, and comparative affinity measurements of protein–ligand interactions, while providing a viable route for developing high-performance, environmentally friendly arsenic adsorbents.

## Materials and methods

### Phylogeny and multiple sequence alignment of ArsRs

Phylogenetic analysis was used to infer the evolutionary relationships among the ArsRs proteins of various organisms. Acquisition of ArsR homologous sequence and conserved arsenic binding domain was performed by searching from the National Center for Biotechnology Information (NCBI) protein database using a BLASTP search. The phylogenetic tree was constructed by the Neighbor-joining method (NJ) using MEGA 7.0. The statistical significance of the branch pattern was estimated by conducting a 1,000 bootstraps. The amino acid sequences of nine RpArsRs derived from *R. palustris* CGA009 were aligned with each other using Clustal X2.

### Strain construction fused with R_*P*_arsRs-SpyTag and Rfp reporter genes

*R. palustris* CGA009 (BAA-98) was obtained from the American Type Culture Collection (ATCC). *E. coli* BL21 (pET-28a, Kan*^R^*), *E. coli* BL21 (pET-22b, fused with ELP-SpyCatcher Tag, Amp*^R^*), *E. coli* (pET28a, fused with His-*mrfp*-SpyTag, Kan*^R^*), *E. coli* (pET28a, fused with His-*rparsR*-SpyTag, Kan*^R^*), *E. coli* TOP10 (pUC19, fused with T7-*mrfp*, Amp*^R^*) are constructed and preserved in our lab. The *RparsRs* genes (encoding RpArsR protein) and *mrfp* gene (encoding red fluorescence protein, mRFP) were amplified from *R. palustris* CGA009 and *E. coli* TOP10, respectively. For simplifying protein purification process, we constructed engineered *E. coli* strains expressing fusion proteins containing an elastin-like polypeptide (ELP) tag, His-tag, and SpyTag (*hisTag*-*rparsRs*/*mrfp*-*spyTag*).

Strain CGA009 was cultured anaerobically in modified Ormerod’s liquid medium at 30°C under continuous illumination (2,000 lux) using screw-capped bottles. For recombinant protein expression, *E. coli* BL21 (pET-22b-ELPs-SpyCatcher) was grown in LB medium supplemented with 50 μg/mL ampicillin, incubated aerobically at 37°C with 200 rpm shaking in the dark. BL21(pET28a) was grown in Terrific Broth (12 g/L tryptone, 24 g/L yeast extract, 30 g/L ammonium chloride, 4.5 mL/L glycerol) containing 50 μg/mL kanamycin, grown at 37°C with 200 rpm shaking until reaching OD_600_ 0.5–0.6. All recombinant strains were induced with 0.2 mM isopropyl β-D-1-thiogalactopyranoside (IPTG) at 16°C for 24 h prior to protein extraction. The primers used for plasmid constructions are listed in Supplementary Table 1.

### RpArsRs preparation

Protein crude extract was prepared as follows: bacterial cells were harvested by centrifugation at 10,000 rpm for 5 min. The cell pellets were resuspended in precooling 0.1 M of PBS solution (1.136 g/L NaH_2_PO_4_, 7.888 g/L NaCl, 0.272 g/L KH_2_PO_4_, 0.1973 g/L KCl, pH 7.4) and disrupted by ultrasonication (350 W output, pulse 4 s, stop 4 s) for 20 min in an ice bath followed by centrifugation at 4°C and 12,000 rpm for 20 min, and the supernatant containing the soluble protein fraction was collected for subsequent experiments. The fusion protein of ELP-SpyCatcher was purified by using a non-chromatographic method termed as the inverse transition cycle (ITC) (Ke et al., 2019; Lin et al., 2019). The protein concentration of ELP-SpyCatcher was calculated by Tryptophan standard curve of each standard substance. The fusion protein of RpArsR/RFP-SpyTag was purified by using Ni-NTA affinity chromatography. Following crude extract preparation, the cell lysate was filtered through a 0.45 μm sterile membrane and applied to a Ni-NTA column pre-equilibrated with binding buffer (50 mM Na_2_HPO_4_, 300 mM NaCl, 10 mM Tris base, pH 8.0). Target proteins were then eluted using a step gradient of imidazole (100 mM, 150 mM and 200 mM) in pH 7.0 PBS buffer. Protein purity was assessed by 12% sodium dodecyl sulfate polyacrylamide gel electrophoresis (SDS-PAGE), and the RpArsR concentration was quantified using the Bradford method with Coomassie Brilliant Blue G-250.

### Synthesis of biosilica spheres (ELP-SpyCatcher@SiO_2_, abbreviated as S)

A fresh orthosilicic acid solution was prepared by dissolving 1.522 g of tetramethyl orthosilicate (TMOS) in 7 mL of 1 mM HCl, then diluted to a final volume of 10 mL. The solution was equilibrated at room temperature for 10 min before use. Purified ELPs-SpyCatcher was dissolved in 0.1 mM HCl at a final concentration of 100 μM. ELPs-SpyCatcher and orthosilicic acid solutions were mixed at a 9:1 (v:v) ratio for 10 min. The resulting biosilica spheres (S) were collected by centrifugation at 5,000 rpm, 4°C for 5 min and washed three times with deionized water. The concentration of S (calculated by SiO_2_) was determined using silicon molybdenum yellow spectrophotometry (Lechner and Becker, 2014). The mass concentration of suspension of biosilica spheres (S) was adjusted to 1.00 g/L with 0.1M PBS buffer (pH 7.4). The relationship of unit mass (g) and the particles number (n) per unit volume (L) was measured by microscopic counting method. The molar mass of S was calculated using Avogadro constant (6.02 × 10^23^/M).

### Synthesis and characterization of solid-phase arsenic adsorbent (S-RpArsR)

To construct the solid-phase arsenic adsorbent (S-RpArsR), RpArsR-SpyTag fusion proteins were immobilized onto ELP-SpyCatcher@SiO_2_ biosilica spheres via the SpyCatcher/SpyTag system, which enables specific and covalent self-assembly. As a control for evaluating immobilization efficiency and dispersion, a red fluorescent protein (RFP)-SpyTag fusion was similarly immobilized onto biosilica spheres to produce S-RFP. For preparation of non-purified S-RpArsR, biosilica spheres (ELP-SpyCatcher@SiO_2_) were incubated directly with crude RpArsR-SpyTag protein extracts, and S-RpArsR complexes were collected by centrifugation at 12,000 rpm for 5 min. The multiple round immobilizations of RpArsR were performed by resuspending S-RpArsR in fresh crude RpArsR-SpyTag extract and repeating the centrifugation step. Unless otherwise noted, all references to “S-RpArsR” in this study refer to samples prepared through a single immobilization cycle.

It should be noted that purified RpArsR-SpyTag proteins were exclusively used for initial methodological validation (e.g., determining maximum theoretical loading and baseline *K*_A_ comparisons) to establish system reliability. All subsequent analyses of the nine homologs, as well as preparation of functional adsorbents, were performed using the purification-free (crude lysate) strategy. To prepare S-RpArsR2 conjugates, 2.2 μM RpArsR2-SpyTag was incubated with varying concentrations of biosilica spheres for 10 min in a 0.1 M PBS buffer (pH 7.4). The immobilized S-RpArsR2 concentration was calculated by subtracting the free RpArsR2 concentration in the supernatant from the initial protein concentration. The maximum RpArsR2 loading capacity per mole of biosilica spheres was determined by plotting the bound RpArsR2 concentration against the molar concentration of biosilica spheres and performing linear regression on the linear segment. The slope of the fitted equation represents the theoretical maximum immobilization capacity. For morphological and elemental characterization, S-RpArsR2 samples were treated with arsenic, washed three times with deionized water, and dried overnight on silicon wafers.

### Quantitative analysis of RpArsR-As(III) binding affinity

To quantify As(III) binding, 2.753 × 10^–7^ μM of S-RpArsR2 was incubated with 1.00 g/L of or 100 μg/L of As(III) (1.33 μM) in pH 7.0 TB74s buffer (50 mM Tris-HCl, 150 mM NaCl) (Kostal et al., 2004) at 25°C for 12 h, with 220 rpm shaking, The mixture was centrifuged at 10,000 g for 5 min, washed three times with deionized water, then analyzed for As content. All treatments were performed in triplicate. For pH optimization, the pH of TB74s buffer was adjusted with 1 mM HCl or NaOH. Under the optimized pH, As(III) adsorption by S-RpArsR2 was further evaluated by varying incubation times (2–60 min). Then the maximum adsorption capacity, affinity constant (*K*_A_) and the numbers of arsenic binding sites on S-RpArsR2 were subsequently determined. Biosilica spheres (S) without RpArsR2 were used as the control to account for non-specific adsorption.

To evaluate the effect of immobilization on RpArsR2-As(III) binding affinity, the parallel competitive systems were designed as follows, (1) S-RpArsR2 (immobilized) + free RpArsR2 (7.85 μM) + As(III), and (2) S-RpArsR2 + BSA (15.2 μM) + As(III). The fixed proteins and free proteins competed with each other with As(III) in reaction system. After incubation (pH 7.0, 25°C, 12 h, 220 rpm), systems were centrifuged (12,000 rpm, 5 min) and washed, arsenic amount was quantified using atomic fluorescence spectrophotometr, and their affinity capacity were compared.

### Assessment of heavy metal interference on S-RpArsR As(III) adsorption

To evaluate the selectivity of the S-RpArsR for As(III) adsorption, we systematically examined potential interference from six common heavy metal contaminants, Cu(II) (CuSO_4_), Zn(II) (ZnSO_4_), Pb(II) (Pb(NO_3_), Cd(II) (CdCl_2_), Cr(VI) (K_6_Cr_2_O_7_), Ni(II) (NiSO_4_). The competitive adsorption assay was performed by introducing each metal ion at a concentration of 13.3 μM (10-fold higher than the 1.33 μM As(III) in pH 7.0 TB74s containing 1.00 g/L S-RpArsR (2.753 × 10^–7^ μM). The As(III) adsorption efficiency was represented by the ratio of As adsorption in the presence of heavy metals to that in metal-free controls (defined as 100% adsorption capacity), with interference effects calculated as percentage reduction in arsenic binding.

### Phytoremediation application of S-RpArsR

To evaluate the potential of S-RpArsR for arsenic phytoremediation, we conducted hydroponic experiments using *Amaranthus tricolor*. The roots of healthy plant *Amaranthus tricolor* (fresh weight 1.96 ± 0.24 g) were soaked in 0.1% potassium permanganate solution for 10 min for disinfection and rinsed with deionized water and then cultured in nutrition solution within 400 mL of black soilless hydroponic box (11.5 cm × 10.5 cm × 8 cm). Each box cultured six plants. Experiments were divided into six groups: (1) negative control [CK-N, no As(III)], (2) positive control [CK-P, 2.67 μM As(III)], (3–5) three S-RpArsR variants (S-RpArsR2, S-RpArsR3, S-RpArsR8), and (6) 0.1 g/L biosilica spheres (S) as a control. Plants were precultured for 2 d, then As(III) and S were added into box and incubated for 4 d. The root whiskers were cut off and balanced fresh weight of plant, then dried at 105°C for 30 min, 65°C for 12 h and balanced dry weight of plant. Plant water content was calculated as the difference between fresh and dry weight. Dried plant tissues were ground and sieved for arsenic analysis. Arsenic contents in both plant tissues and solid-phase arsenic adsorbents were determined using atomic fluorescence spectrophotometry (Deng et al., 2021). Arsenic accumulation in plants was expressed as arsenic concentration per unit dry weight (mg/g).

### Statistical analysis

The statistical analysis was conducted by using one-way analysis of variance (ANOVA) followed by a multiple comparison (LSD-test). Statistical significance was set at *P* ≤ 0.05.

## Data Availability

The original contributions presented in this study are included in the article/Supplementary material, further inquiries can be directed to the corresponding authors.

## References

[B1] Aguado J. Arsuaga J. M. Arencibia A. Lindo M. Gascon V. (2009). Aqueous heavy metals removal by adsorption on amine-functionalized mesoporous silica. *J. Hazard. Mater.* 163 213–221. 10.1016/j.jhazmat.2008.06.080 18675509

[B2] Andres J. Bertin P. N. (2016). The microbial genomics of arsenic. *FEMS Microbiol. Rev.* 40 299–322. 10.1093/femsre/fuv050 26790947

[B3] Cai L. Liu X. Qiu Y. Liu M. Zhang G. (2020). Enzymatic degradation of algal 1,3-xylan: from synergism of lytic polysaccharide monooxygenases with beta-1,3-xylanases to their intelligent immobilization on biomimetic silica nanoparticles. *Appl. Microbiol. Biotechnol.* 104 5347–5360. 10.1007/s00253-020-10624-w 32318768

[B4] Chen J. Nadar V. S. Rosen B. P. (2017). A novel MAs(III)-selective ArsR transcriptional repressor. *Mol. Microbiol.* 106 469–478. 10.1111/mmi.13826 28861914 PMC5653410

[B5] Chen J. Sarkarai Nadar V. Zhang J. Viswanathan T. Rosen B. P. (2025). Evolutionary advantages of multiple arsenic binding sites in an ArsR transcriptional repressor. *Environ. Sci. Technol.* 59 23530–23541. 10.1021/acs.est.5c11929 41115192

[B6] Chen J. Sun G. X. Wang X. X. Lorenzo V. Rosen B. P. Zhu Y. G. (2014). Volatilization of arsenic from polluted soil by *Pseudomonas putida* engineered for expression of the arsM Arsenic(III) S-adenosine methyltransferase gene. *Environ. Sci. Technol.* 48 10337–10344. 10.1021/es502230b 25122054 PMC4151780

[B7] Chen Y. Rosen B. P. (1997). Metalloregulatory properties of the ArsD repressor. *J. Biol. Chem.* 272 14257–14262. 10.1074/jbc.272.22.14257 9162059

[B8] Deng X. Chen B. Chen Y. Lu L. Yuan X. Yang Y. et al.. (2021). Variations in root morphological indices of rice (*Oryza sativa* L.) induced by seedling establishment methods and their relation to arsenic accumulation in plant tissues. *Environ. Pollut.* 281:116999. 10.1016/j.envpol.2021.116999 33799206

[B9] Dunivin T. K. Yeh S. Y. Shade A. (2019). A global survey of arsenic-related genes in soil microbiomes. *Bmc. Biol.* 17:45. 10.1186/s12915-019-0661-5 31146755 PMC6543643

[B10] Ghahghaei M. Shahpiri A. (2025). Enhanced arsenic accumulation in engineered *Pseudomonas putida* via heterologous expression of a DNA-binding transcription repressor ArsR. *Chemosphere* 387:144659. 10.1016/j.chemosphere.2025.144659 40857961

[B11] Hui C. Y. Liu M. Q. Guo Y. (2024). Synthetic bacteria designed using *ars* operons: a promising solution for arsenic biosensing and bioremediation. *World J. Micro. Biotechnol.* 40:192. 10.1007/s11274-024-04001-2 38709285

[B12] Hussain A. Kabir M. (2026). Synergistic nanobioremediation: exploring nanoparticle-plant-microbe interactions for sustainable soil decontamination. *Nanotechnol. Environ. Eng.* 11:11.

[B13] Jiang G. Gong Z. Li X. F. Cullen W. R. Le X. C. (2003). Interaction of trivalent arsenicals with metallothionein. *Chem. Res. Toxicol.* 16 873–880. 10.1021/tx034053g 12870890

[B14] Kang Y. S. Brame K. Jetter J. Bothner B. B. Wang G. Thiyagarajan S. et al.. (2016). Regulatory activities of four ArsR proteins in *Agrobacterium tumefaciens* 5A. *Appl. Environ. Microbiol.* 82 3471–3480. 10.1128/AEM.00262-16 27037117 PMC4959165

[B15] Ke C. Xiong H. Zhao C. Zhang Z. Zhao X. Rensing C. et al.. (2019). Expression and purification of an ArsM-elastin-like polypeptide fusion and its enzymatic properties. *Appl. Microbiol. Biotechnol.* 103 2809–2820. 10.1007/s00253-019-09638-w 30666362

[B16] Ke C. Zhao C. Rensing C. Yang S. Zhang Y. (2018). Characterization of recombinant *E. coli* expressing *arsR* from *Rhodopseudomonas palustris* CGA009 that displays highly selective arsenic adsorption. *Appl. Microbiol. Biotechnol.* 102 6247–6255. 10.1007/s00253-018-9080-8 29789881

[B17] Kostal J. Yang R. Wu C. H. Mulchandani A. Chen W. (2004). Enhanced arsenic accumulation in engineered bacterial cells expressing ArsR. *Appl. Environ. Microbiol.* 70 4582–4587. 10.1128/AEM.70.8.4582-4587.2004 15294789 PMC492386

[B18] Lechner C. C. Becker C. F. (2014). A sequence-function analysis of the silica precipitating silaffin R5 peptide. *J. Pept. Sci.* 20 152–158. 10.1002/psc.2577 25975421

[B19] Lin Y. Jin W. Qiu Y. Zhang G. (2019). Programmable stimuli-responsive polypeptides for biomimetic synthesis of silica nanocomposites and enzyme self-immobilization. *Int. J. Biol. Macromol.* 134 1156–1169. 10.1016/j.ijbiomac.2019.05.159 31128196

[B20] Maleki F. Shahpiri A. (2022). Efficient and specific bioaccumulation of arsenic in the transgenic *Escherichia coli* expressing ArsR1 from *Corynebacterium glutamicum*. *Biometals.* 35 889–901. 10.1007/s10534-022-00412-6 35767097

[B21] Paez-Espino A. D. Durante-Rodriguez G. de Lorenzo V. (2015). Functional coexistence of twin arsenic resistance systems in *Pseudomonas putida* KT2440. *Environ. Microbiol.* 17 229–238. 10.1111/1462-2920.12464 24673935

[B22] Podgorski J. Berg M. (2020). Global threat of arsenic in groundwater. *Science* 368 845–850. 10.1111/1462-2920.12464 32439786

[B23] Rawle R. A. Kang Y. S. Bothner B. Wang G. McDermott T. R. (2019). Transcriptomics analysis defines global cellular response of *Agrobacterium tumefaciens* 5A to arsenite exposure regulated through the histidine kinases PhoR and AioS. *Environ. Microbiol* 21 2659–2676. 10.1111/1462-2920.14577 30815967

[B24] Rawle R. Saley T. C. Kang Y. S. Wang Q. Walk S. Bothner B. et al.. (2021). Introducing the ArsR-regulated arsenic stimulon. *Front. Microbiol.* 12:630562. 10.3389/fmicb.2021.630562 33746923 PMC7965956

[B25] Tian J. Jia R. Wenge D. Sun H. Wang Y. Chang Y. et al.. (2021). One-step purification and immobilization of recombinant proteins using SpyTag/SpyCatcher chemistry. *Biotechnol. Lett.* 43 1075–1087. 10.1007/s10529-021-03098-x 33591462

[B26] Yang J. B. Rawat S. Stemmler T. L. Rosen B. P. (2010). Arsenic binding and transfer by the ArsD As(III) Metallochaperone. *Biochemistry* 49 3658–3666. 10.1021/bi100026a 20361763 PMC2920133

[B27] Zhang Y. Wu W. Huang K. Zhao F. J. (2025). A new type of ArsR transcriptional repressor controls transcription of the arsenic resistance operon of *Arsenicibacter rosenii* SM-1. *mLife* 4 96–100. 10.1002/mlf2.12155 40026573 PMC11868830

[B28] Zhao C. Zhang Y. Chan Z. Chen S. Yang S. (2015). Insights into arsenic multi-operons expression and resistance mechanisms in *Rhodopseudomonas palustris* CGA009. *Front. Microbiol.* 6:986. 10.3389/fmicb.2015.00986 26441915 PMC4585019

[B29] Zhu Y. G. Yoshinaga M. Zhao F. J. Rosen B. P. (2014). Earth abides arsenic biotransformations. *Annu. Rev. Earth and Planet Sci.* 42 443–467. 10.1146/annurev-earth-060313-054942 26778863 PMC4712701

