# Supplementary material for: Quantifying arsenic-binding affinities of ArsR proteins via biomimetic self-assembly
Source: Front Microbiol. 2026 May 22;17:1846435. doi: 10.3389/fmicb.2026.1846435 (PMC13236881; doi:10.3389/fmicb.2026.1846435)
Supplement: Supplementary file 1 [file Data_Sheet_1.docx]

**Quantifying Arsenic-Binding Affinities of ArsR Proteins via Biomimetic Self-Assembly to Reveal High As Resistance Mechanism**

**Table S1**

Primers used in this study.

| **Gene names** | **Primer sequence (5'→3')** |
| --- | --- |
| *RparsR*1 | F: CGCGGATCCATGGATAACGATTCGGCCATCG |
|  | R: CCGGAATTCTCAGCAGCAGGGTGTGAGTTC |
| *RparsR*2 | F: CGACGCGTATGGAAGCAAGTGTAGCCCTTGA |
|  | R: CGAGCTCGACATCGACGGTCTCTTTCGAA |
| *RparsR*3 | F: CGCGGATCCATGGATAACGATTCGGCCATCG |
|  | R: CCGGAATTCTCATGCAGCCCTCGCGC |
| *RparsR*4 | F: CGCGGATCCATGACCGACACCGCCGAC |
|  | R: CCGGAATTCTCAGTGGGCATTGCGGTTCT |
| *RparsR*5 | F: CGCGGATCCATGACCGACACCGCCGA |
|  | R: CCGGAATTCTCAAGCTTTTTCGTCGGTTTATACG |
| *RparsR*6 | F: CGCGGATCCATGGCAGCCGCGAGCGGTGTTCGGA |
|  | R: CCGGAATTCTCGTCGGTTTATACGCATCAACCAAC |
| *RparsR*7 | F: CGCGGATCCATGAAAATAACAGACGATCCGATGGT |
|  | R: CCGGAATTCTCATGCCCCTGCAGGTTTG |
| *RparsR*8 | F: CGCGGATCCATGGTGCAGTTCGTTCATCCC |
|  | R: CCGGAATTCCTACTCGTCCGCGTGTTTCAG |
| *RparsR*9 | F: CGCGGATCCATGGATGAGGTCTTCAAAGCGCT |
|  | R: CCGGAATTCCTCATCGCGCGTCTCCAG |
| *rfp* | F: CCGGATCCATGGCTTCCTCCGAAGACGTTATC |
|  | R: ACGGAATTCAGCACCGGTGGAGTGAC |

GGATCC and GAATTC were *BamH* I and *EcoR* I recognition site, respectively.

**Table S2**

RpArsRs information of *Rhodopseudomonas palustris* CGA009.

| **Name** | **Accession** | **Lucus tag** | **Product** | **size** | **Protein ID** | ***ars* operon** |
| --- | --- | --- | --- | --- | --- | --- |
| RpArsR1 | CAE 27697.1 | TX73_RS11670 | WP_011157809.1 | 110 | WCL92419.1 | *arsR1C1BH* |
| RpArsR2 | CAE 28997.1 | TX73_RS18430 | WP_011159096.1 | 118 | WCL93732.1 | *arsR2C2C3acr3* |
| RpArsR3 | CAE 29000.1 | TX73_RS18445 | WP_011159099.1 | 99 | WCL93735.1 | - |
| RpArsR4 | CAE 29002.1 | TX73_RS18455 | WP_011159101.1 | 118 | WCL93737.1 | *arsR4M* |
| RpArsR5 | CAE 29915.1 | TX73_RS23230 | WP_011160008.1 | 124 | WCL94675.1 | - |
| RpArsR6 | CAE 26440.1 | TX73_RS05130 | WP_011156530.1 | 142 | WCL91128.1 | - |
| RpArsR7 | CAE 26462.1 | TX73_RS05240 | WP_234803362.1 | 113 | WCL91150.1 | - |
| RpArsR8 | CAE 27602.1 | TX73_011145 | WP_013503052.1 | 104 | WCL92314.1 | - |
| RpArsR9 | CAE 26725.1 | TX73_RS06580 | WP_011156846.1 | 104 | WCL91417.1 | - |

**Table S3**

Linear fitting equations of [R]_f_/[R]_b_ plot vs [P]_f_/[P]_b_ and *K*_A_ of free proteins.

| Competitive systems | Equation | R^2^ | K_D1_/K_D2_ | SD(K_D1_/K_D2_) | *K*_A_×10^6^ | SD(*K*_A_) x10^6^ |
| --- | --- | --- | --- | --- | --- | --- |
| S-RpArsR2 vs RpArsR2 | y = 1.0256x - 0.0169 | 0.9989 | 1.0256 | 0.01396 | 31.28 | 0.0414 |
| S-RpArsR2 vs BSA | y = 0.0059x + 0.2187 | 0.9932 | 0.0059 | 4.27113E-4 | 0.180 | 0.0078 |

**Table S4**

Affinity constants (*K*_A_) and binding site numbers (n) of between BSA-As interaction.

| T(K) | *K*_A_(M) | n | Fitting equation | R^2^ |
| --- | --- | --- | --- | --- |
| 298 | 1.39×10^5^ | 1.07 | y = 1.0742x + 5.1422 | 0.9824 |


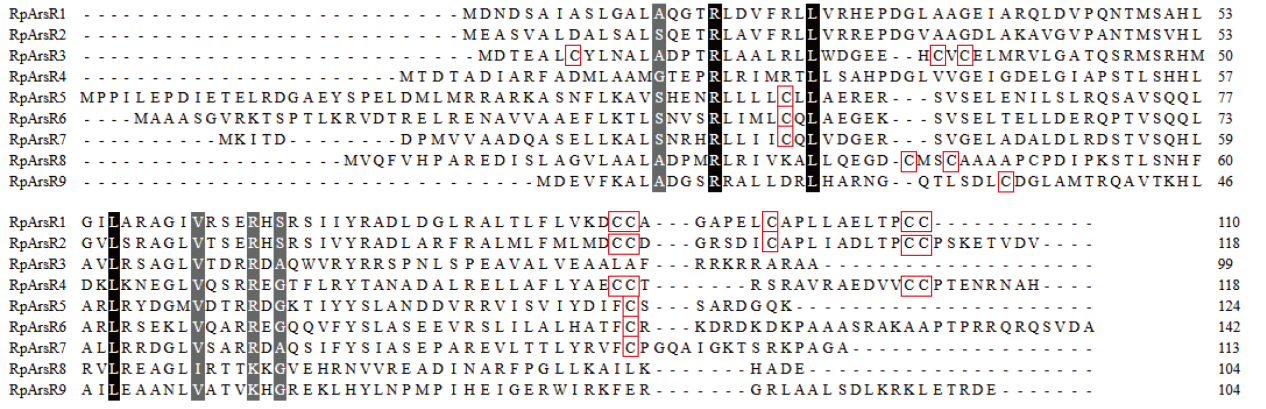


Fig. S1. Multiple alignments of nine RpArsRs from *R. palustris* CGA009.

**
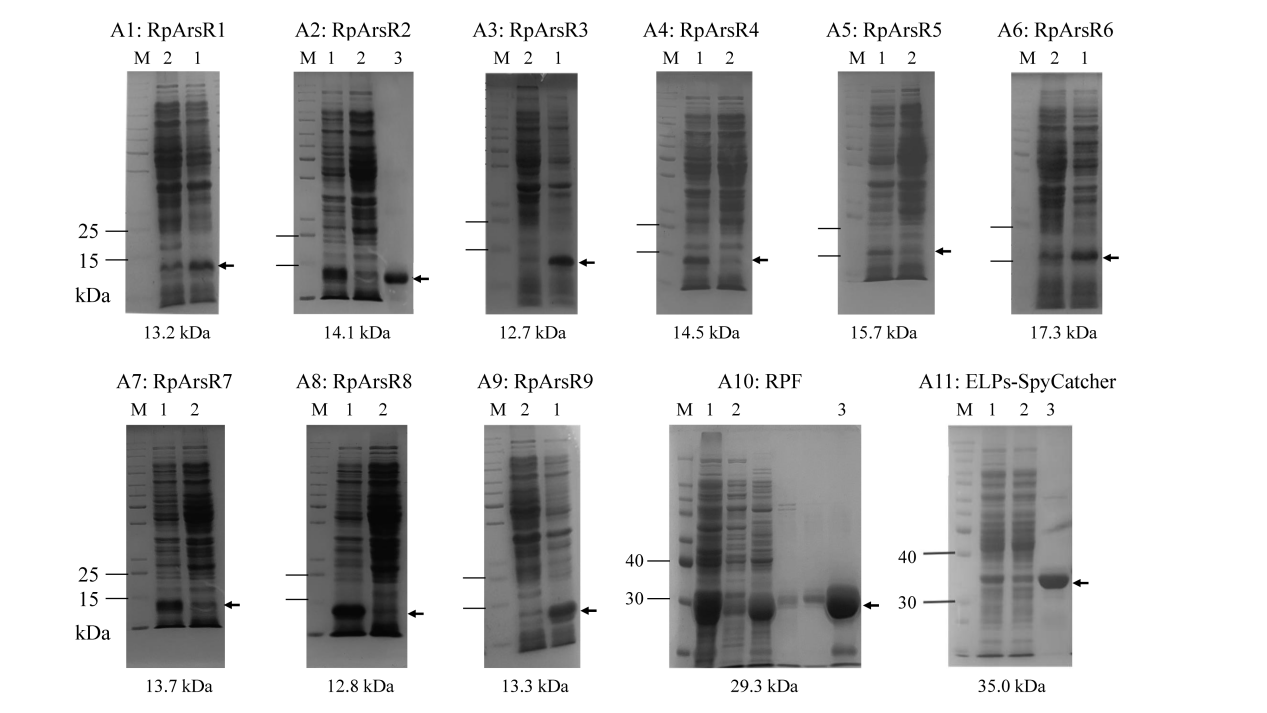
**

Fig. S2. Purification of RpArsRs-SpyTag, RFP-SpyTag and ELPs-SpyCatcher proteins.

**
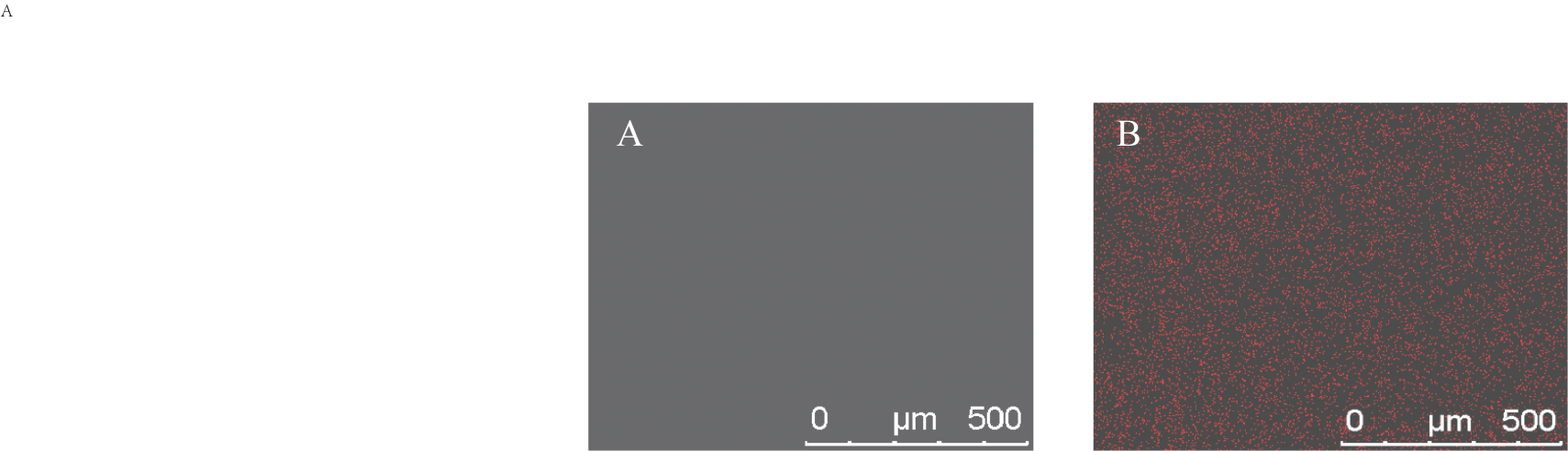
**

Fig. S3. Dispersion of S-RFP by laser confocal microscopy.


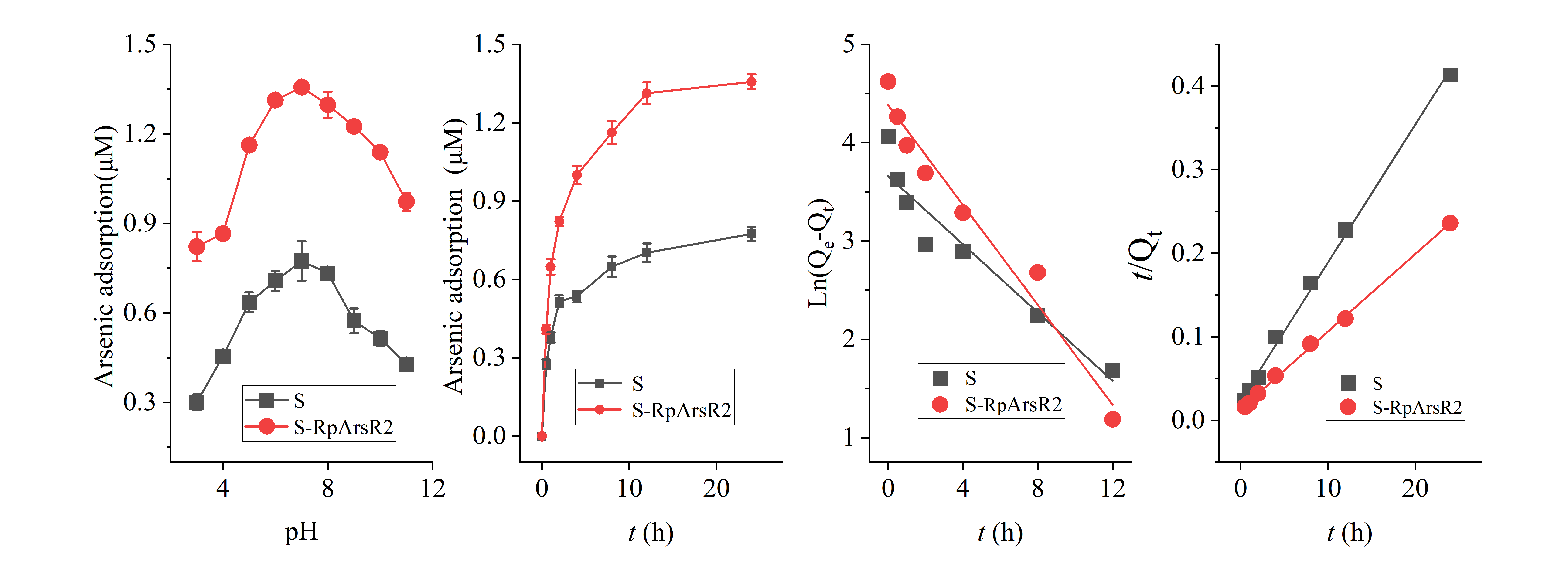


Fig. S4. Kinetics of As(III) Adsorption by S‑RpArsR2.

**
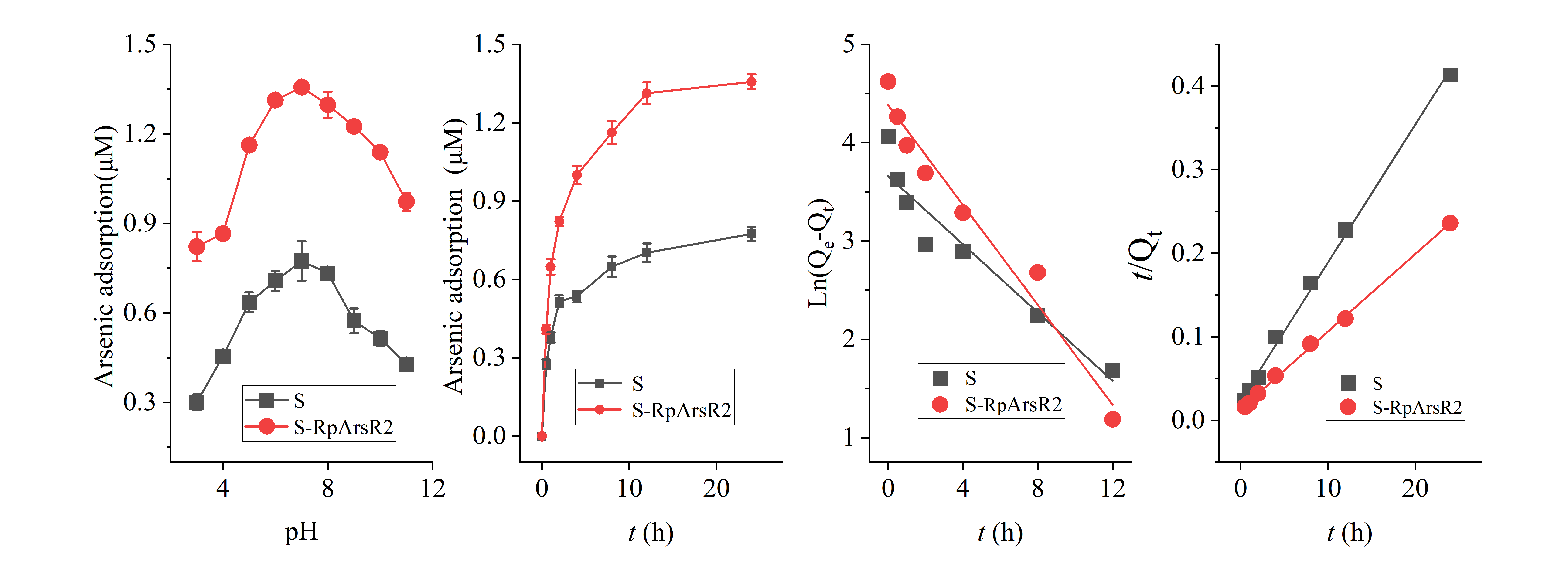
**

Fig. S5. Curves of fluorescence quenching (A) and linear fitting between BSA-As interaction (B).


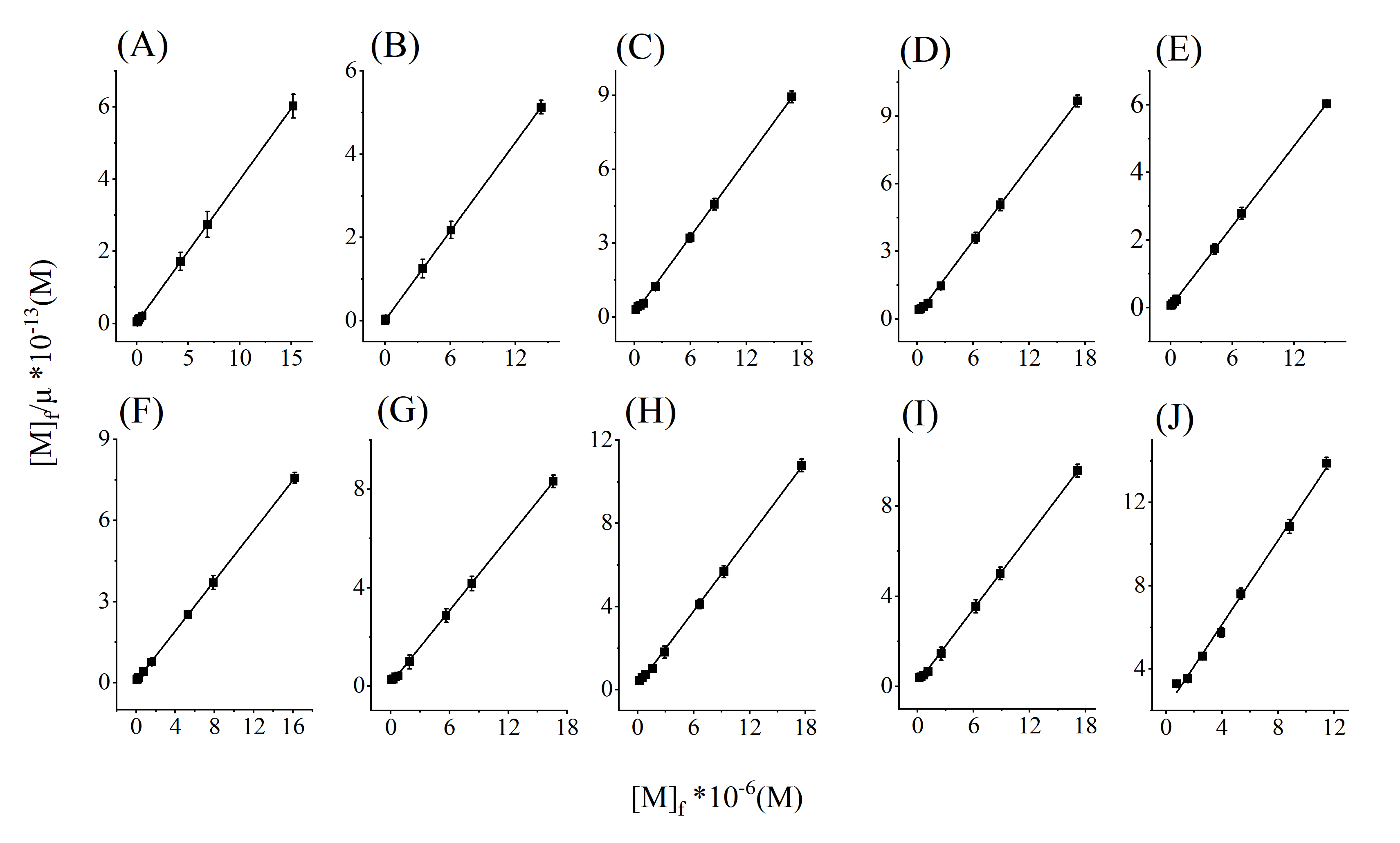
Fig. S6. Scatchard plots of nine S-RpArsRs to As (III) adsorption.

**
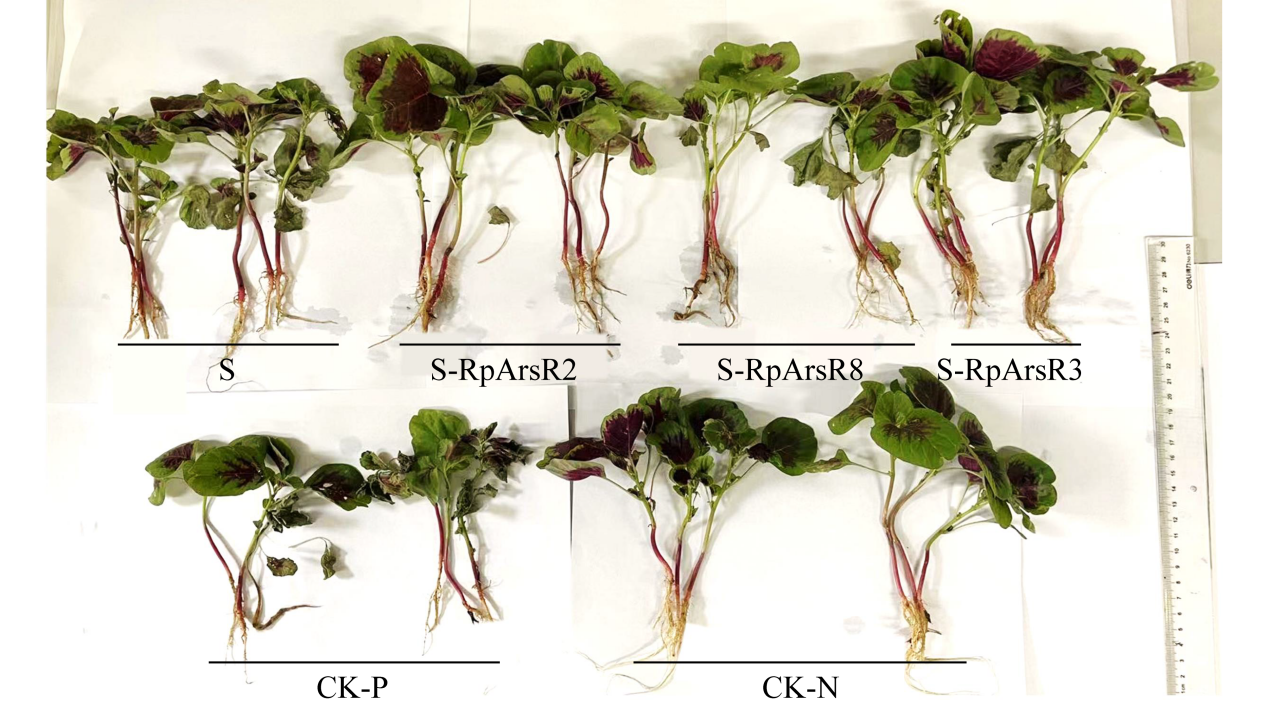
**

Fig. S7 Alleviation of arsenic toxicity in *Amaranthus tricolor* by S-RpArsRs treatments. CK-N represents the negative control (no As(III) stress); CK-P represents the positive control (2.67 μM As(III) stress only); S represents the biosilica-only control (biosilica spheres + 2.67 μM As(III)).


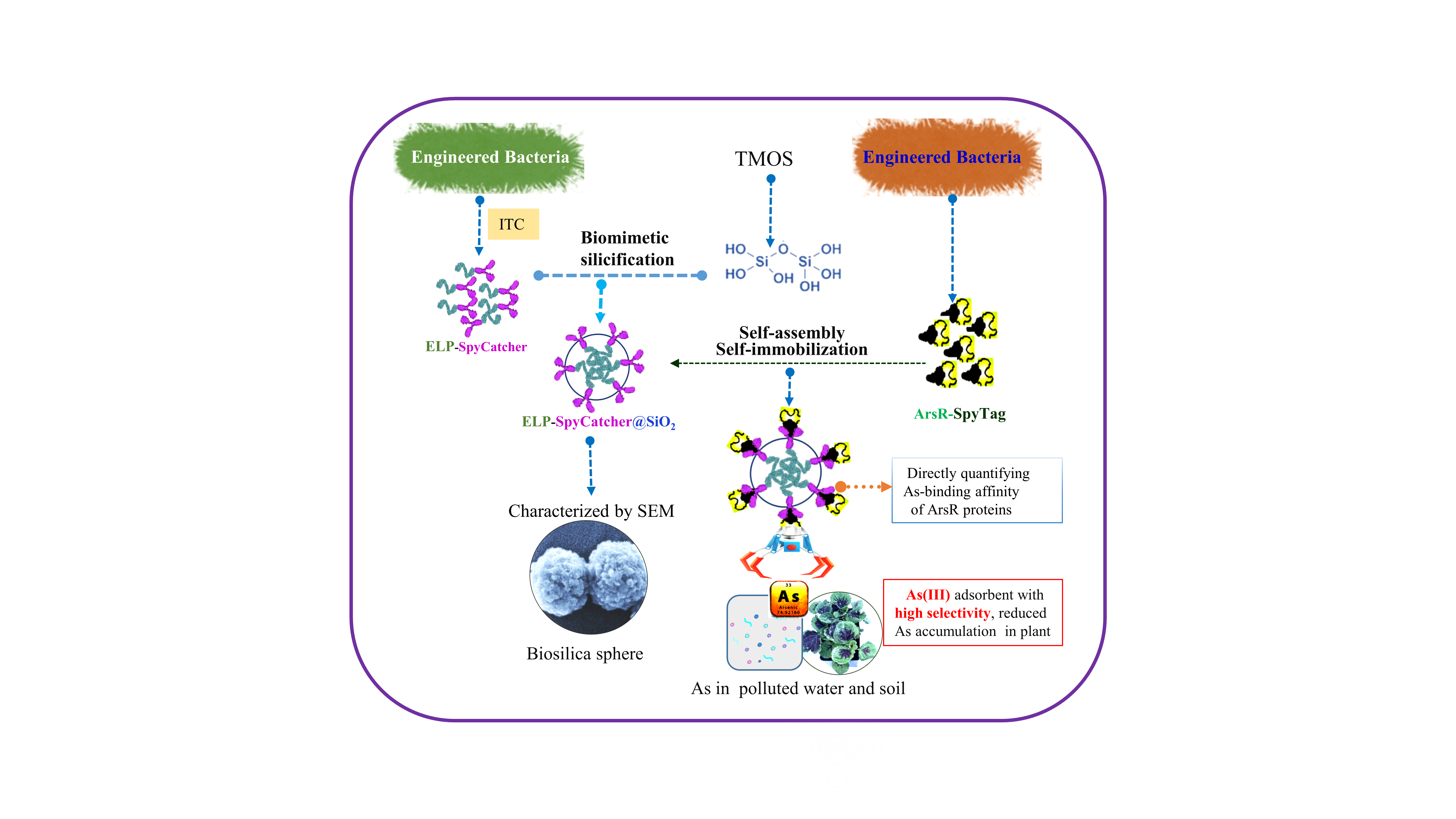


Fig. S8 Schematic diagram of experimental design and workflow.
